# Supplementary material for: Multi-modal dissection of cell-type specific TDP-43 pathology in the motor cortex
Source: Nat Commun. 2026 Mar 9;17:2406. doi: 10.1038/s41467-026-69944-6 (PMC12982666; doi:10.1038/s41467-026-69944-6)
Supplement: Supplementary file 1 — Supplementary Information [file 41467_2026_69944_MOESM1_ESM.pdf]

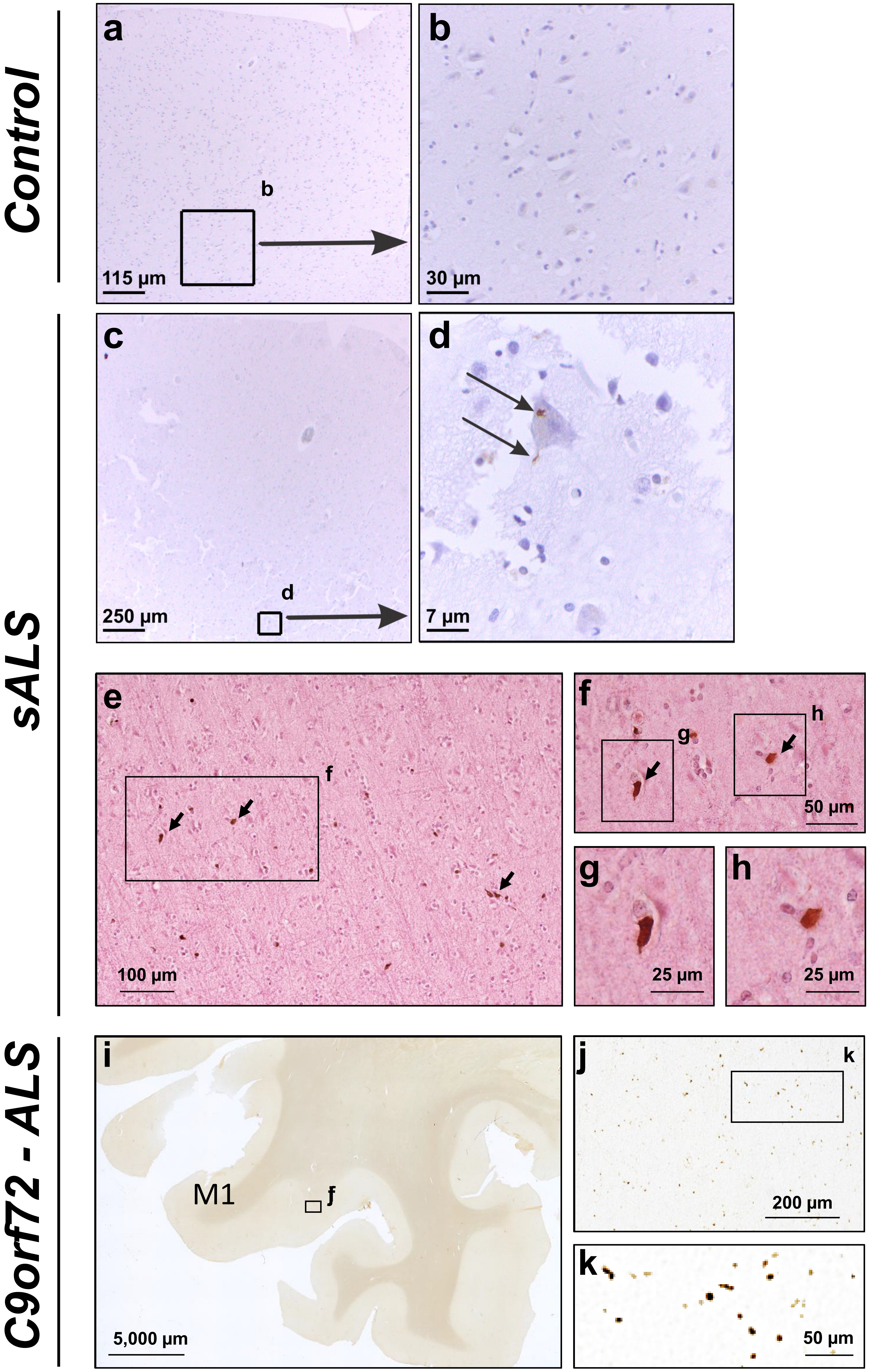

Control
a
b
115 µm
b
30 µm
sALS
c
d
250 µm
d
7 µm
e
f
100 µm
f
g
h
50 µm
g
25 µm
h
25 µm
C9orf72 - ALS
i
M1
f
5,000 µm
j
k
200 µm
k
50 µm

Suppl. Fig. 1. IHC demonstration of pTDP43 aggregates in the frontal/motor cortex of ALS cases. a-d: Examples of pTDP43 inclusions in the frontal cortex (a, b: neurologically unaffected control with absence of pTPD43 inclusions). c, d: sALS case. Arrows point to neuronal pTDP43 inclusions **e-h** Examples of skein-like pTDP43 inclusions in a paraffin section counterstained with H.E. in a sporadic ALS case. Boxed areas are shown at higher magnification in adjacent images. Arrows point to neuronal pTDP43 inclusions. **i-k** Case with *C9ORF72 h*exanucleotide repeat expansion shows a high density of pTDP43 aggregates, as shown in 100 µm-thick hemispheric brain section. Presence of Betz cells was confirmed on the same gyrus in an adjacent section stained with the pigment Nissl stain (not shown). Scale bars are shown in each image/inset.

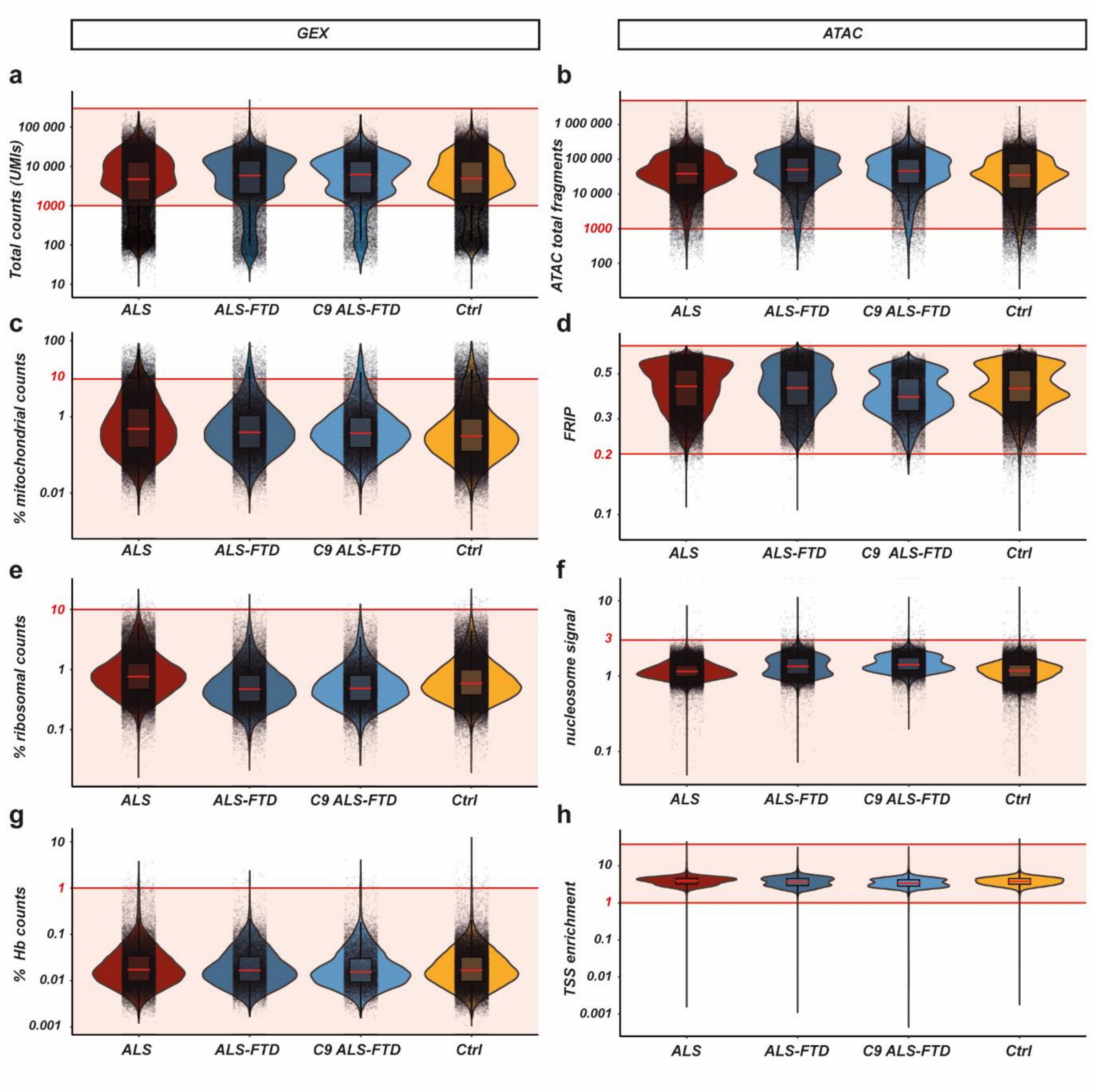

Suppl. Fig. 2. *In silico* nuclei filtering for the generation of the multimodal ALS-FTD motor cortex single-nuclei dataset. Multi-modal nuclei profiles were filtered for quality control to retain only high-quality nuclei in the fully merged (i.e., all samples) dataset. Nuclei were filtered based on quality control thresholds in both modalities, GEX and ATAC-seq per nucleus: total number of unique UMIs ('counts', **a**, >1 000 UMIs), percentage of GEX counts in mitochondrial genes (**c**, <10 %), percentage of GEX counts in ribosomal genes (**e**, <10 %), percentage of counts in hemoglobin genes (**g**, <1 %), total number of unique ATAC-seq fragments (**b**, > 1 000), fraction of reads in ATAC-seq peaks ('FRiP', **d**, > 0.2), nucleosome signal (ratio of mononucleosomal to nucleosome-free fragments, **f**, <3), transcriptional start

site (‘TSS’) enrichment (**h**, >1). The red area and red y-axis labels signify the range in which the nuclei were retained after filtering. Box plots with median (red center line), first and third quartiles (25/75%) and minimum/maximum value within 1.5x interquartile range from the first/third quantile, respective (lower/upper whisker).

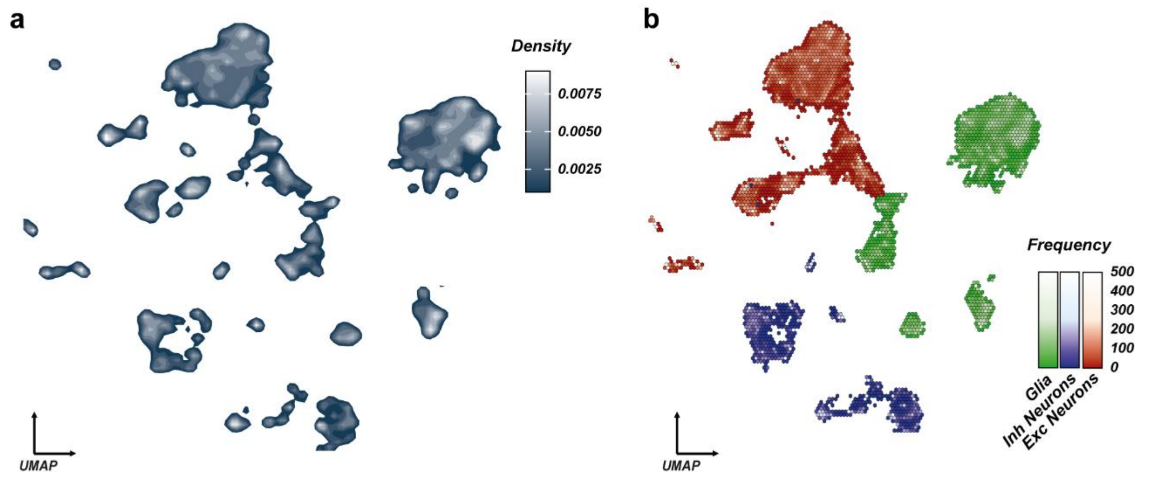

Suppl. Fig. 3. Density plots for the 2-D representation of the multi-omic single-nucleus ALS/ALS-FTD motor cortex dataset. Uniform manifold approximation and projection (UMAP) projections of the dataset were plotted with a density heatmap (**a**) or in hexagonal bins (**b**) to demonstrate the overplotting in this representation. Note that UMAP does not fully preserve local and global structures in the data and heavily distorts distances and relations.

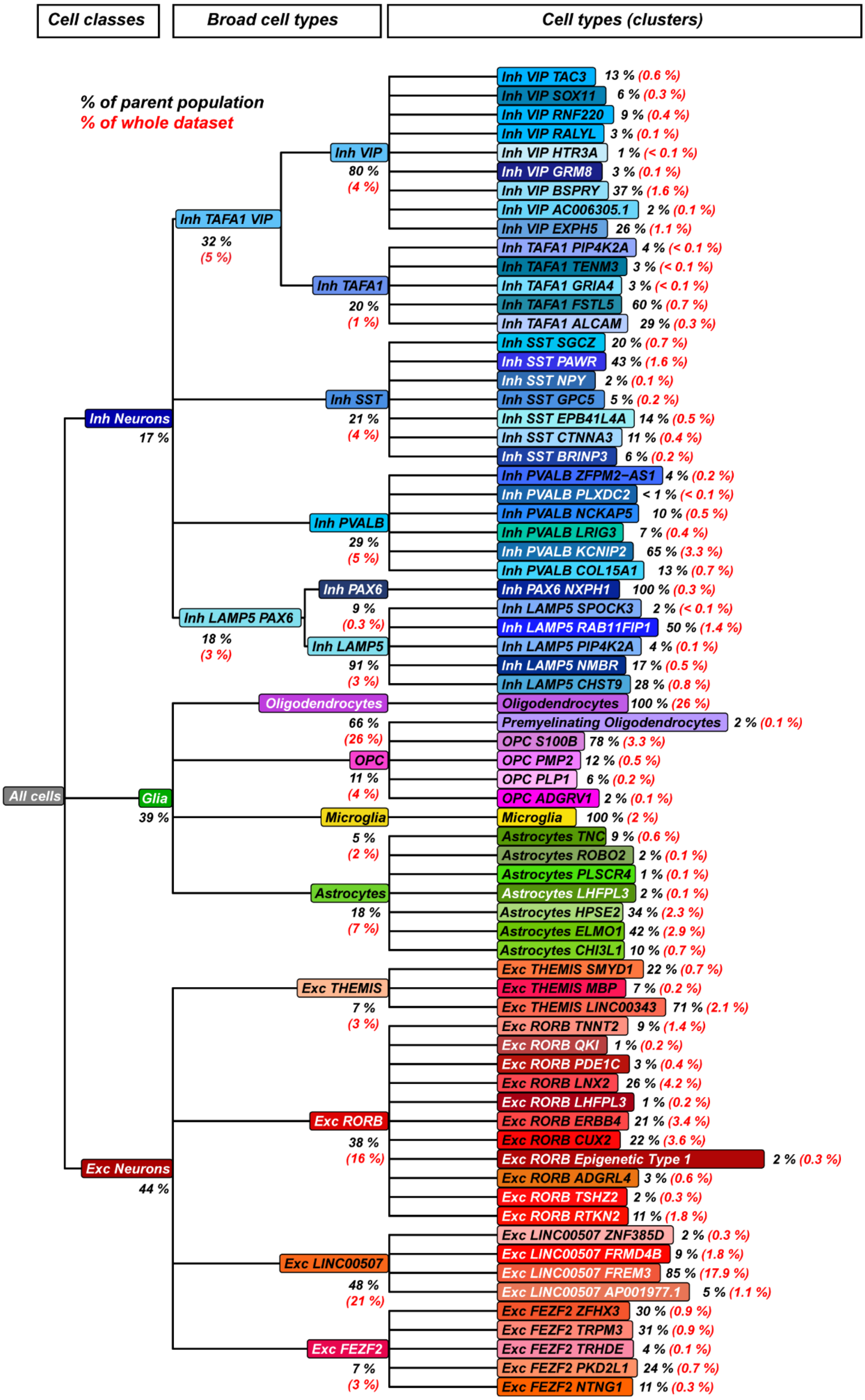

Suppl. Fig.4 Hierarchical cell type annotation. A dendrogram presenting the

hierarchical cell-type annotation based on the expression of known markers, as it was annotated manually. Edge length and node height do not represent distance. Percentages show the percentage of each labeled node from its parent cell population (numbers in black) and from the entire dataset (numbers in red).

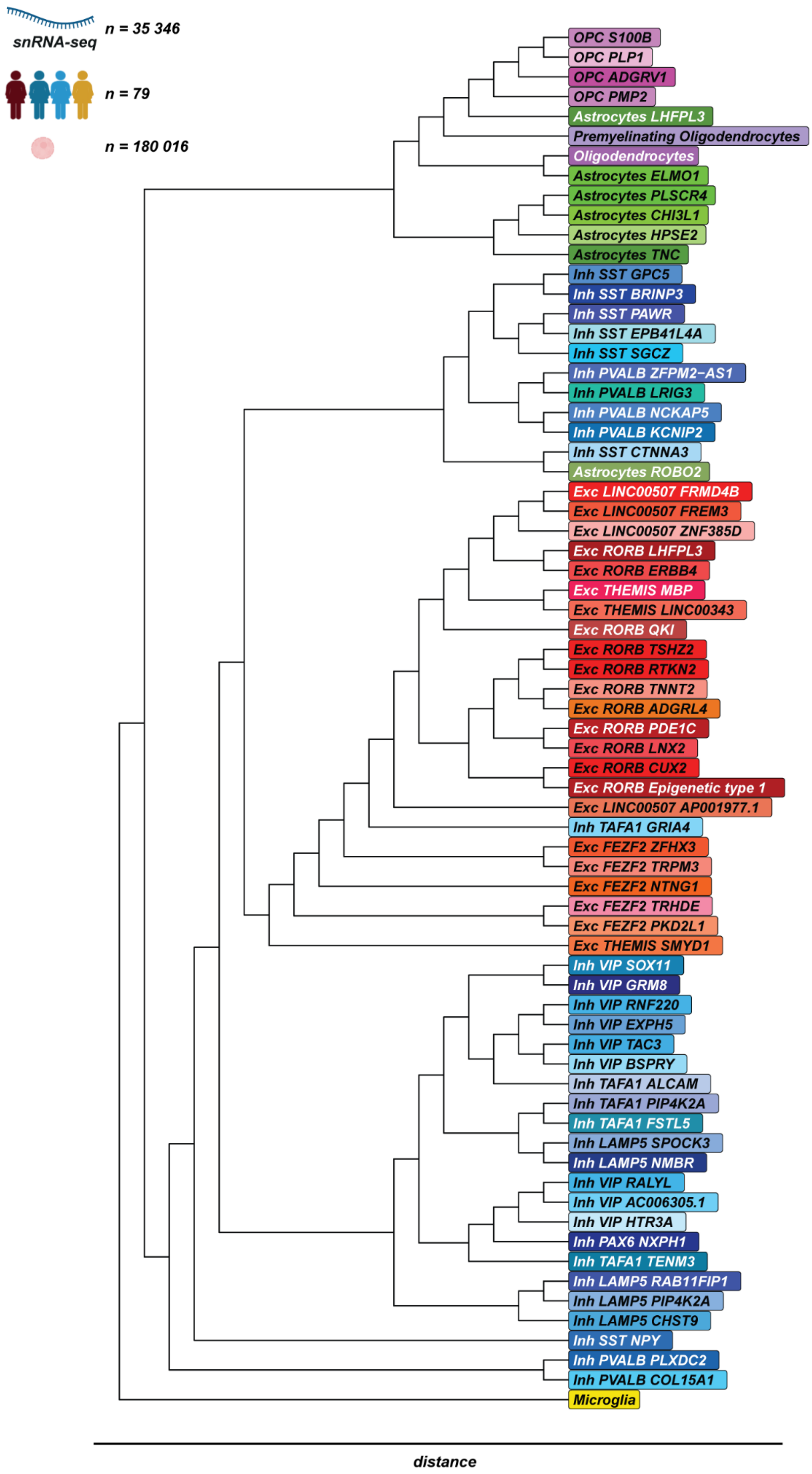
snRNA-seq
n = 35 346
n = 79
n = 180 016
OPC S100B
OPC PLP1
OPC ADGRV1
OPC PMP2
Astrocytes LHFPL3
Premyelinating Oligodendrocytes
Oligodendrocytes
Astrocytes ELMO1
Astrocytes PLSCR4
Astrocytes CHI3L1
Astrocytes HPSE2
Astrocytes TNC
Inh SST GPC5
Inh SST BRINP3
Inh SST PAWR
Inh SST EPB41L4A
Inh SST SGCZ
Inh PVALB ZFPM2−AS1
Inh PVALB LRIG3
Inh PVALB NCKAP5
Inh PVALB KCNIP2
Inh SST CTNNA3
Astrocytes ROBO2
Exc LINC00507 FRMD4B
Exc LINC00507 FREM3
Exc LINC00507 ZNF385D
Exc RORB LHFPL3
Exc RORB ERBB4
Exc THEMIS MBP
Exc THEMIS LINC00343
Exc RORB QKI
Exc RORB TSHZ2
Exc RORB RTKN2
Exc RORB TNNT2
Exc RORB ADGRL4
Exc RORB PDE1C
Exc RORB LNX2
Exc RORB CUX2
Exc RORB Epigenetic type 1
Exc LINC00507 AP001977.1
Inh TAFA1 GRIA4
Exc FEZF2 ZFHX3
Exc FEZF2 TRPM3
Exc FEZF2 NTNG1
Exc FEZF2 TRHDE
Exc FEZF2 PKD2L1
Exc THEMIS SMYD1
Inh VIP SOX11
Inh VIP GRM8
Inh VIP RNF220
Inh VIP EXPH5
Inh VIP TAC3
Inh VIP BSPRY
Inh TAFA1 ALCAM
Inh TAFA1 PIP4K2A
Inh TAFA1 FSTL5
Inh LAMP5 SPOCK3
Inh LAMP5 NMBR
Inh VIP RALYL
Inh VIP AC006305.1
Inh VIP HTR3A
Inh PAX6 NXPH1
Inh TAFA1 TENM3
Inh LAMP5 RAB11FIP1
Inh LAMP5 PIP4K2A
Inh LAMP5 CHST9
Inh SST NPY
Inh PVALB PLXDC2
Inh PVALB COL15A1
Microglia
distance

Suppl. Fig. 5. Hierarchical clustering of the cell types based on the transcriptome. The 70 weighted nearest neighbor cell clusters were hierarchically clustered with L_1 norm (Manhattan distance), which is suggested as more suitable for high-dimension data, and with the average linkage agglomerative clustering algorithm using all detected RNA features *(n=35 346)* and all samples *(n=79)* and cells *(n=180 016)* with log-normalized counts. Icon art generated with BioRender.com (*Created in BioRender. Lee, J. (2026) https://BioRender.com/o64cy0j*).

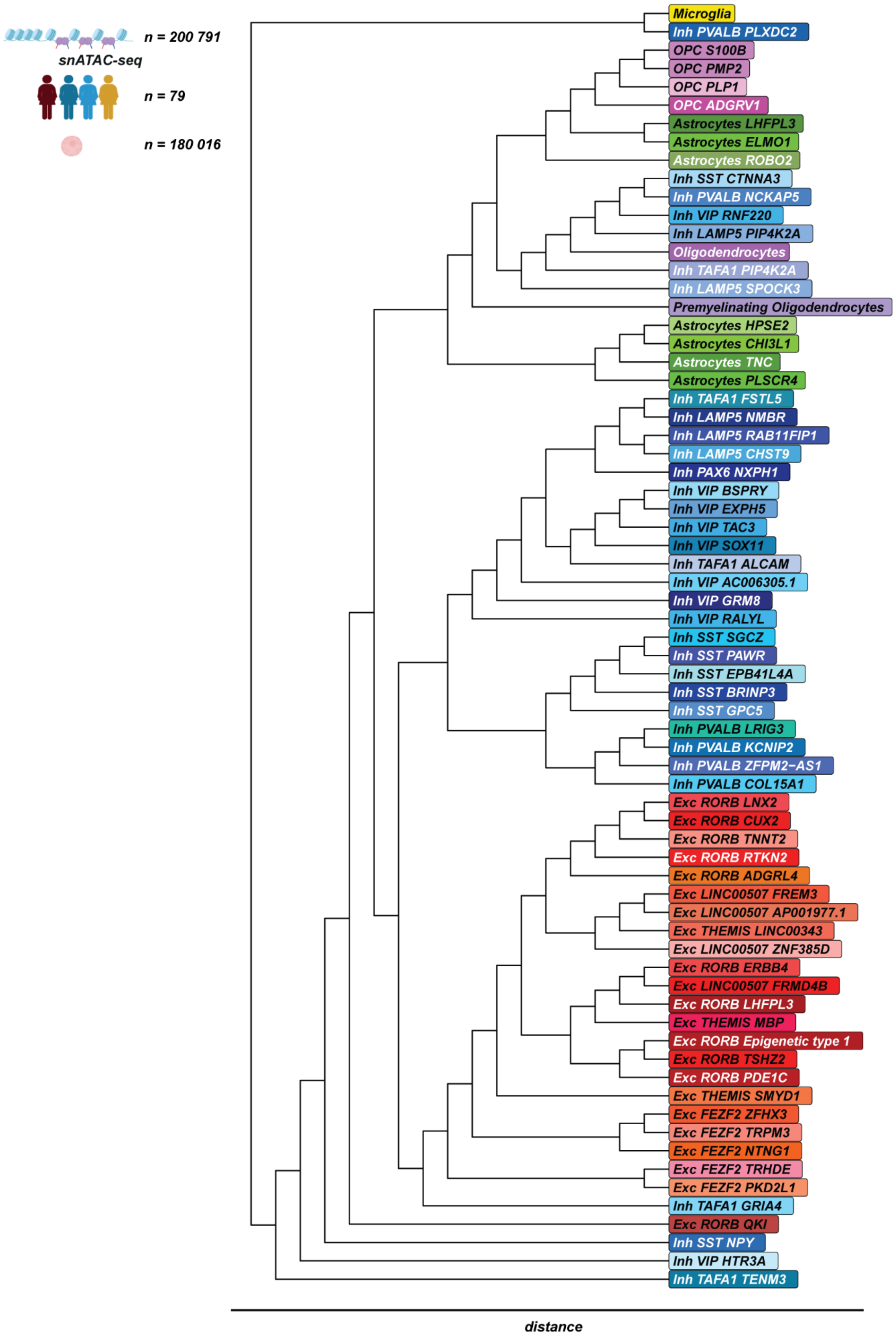
n = 200 791
snATAC-seq
n = 79
n = 180 016
Microglia
Inh PVALB PLXDC2
OPC S100B
OPC PMP2
OPC PLP1
OPC ADGRV1
Astrocytes LHFPL3
Astrocytes ELMO1
Astrocytes ROBO2
Inh SST CTNNA3
Inh PVALB NCKAP5
Inh VIP RNF220
Inh LAMP5 PIP4K2A
Oligodendrocytes
Inh TAFA1 PIP4K2A
Inh LAMP5 SPOCK3
Premyelinating Oligodendrocytes
Astrocytes HPSE2
Astrocytes CHI3L1
Astrocytes TNC
Astrocytes PLSCR4
Inh TAFA1 FSTL5
Inh LAMP5 NMBR
Inh LAMP5 RAB11FIP1
Inh LAMP5 CHST9
Inh PAX6 NXPH1
Inh VIP BSPRY
Inh VIP EXPH5
Inh VIP TAC3
Inh VIP SOX11
Inh TAFA1 ALCAM
Inh VIP AC006305.1
Inh VIP GRM8
Inh VIP RALYL
Inh SST SGCZ
Inh SST PAWR
Inh SST EPB41L4A
Inh SST BRINP3
Inh SST GPC5
Inh PVALB LRIG3
Inh PVALB KCNIP2
Inh PVALB ZFPM2−AS1
Inh PVALB COL15A1
Exc RORB LNX2
Exc RORB CUX2
Exc RORB TNNT2
Exc RORB RTKN2
Exc RORB ADGRL4
Exc LINC00507 FREM3
Exc LINC00507 AP001977.1
Exc THEMIS LINC00343
Exc LINC00507 ZNF385D
Exc RORB ERBB4
Exc LINC00507 FRMD4B
Exc RORB LHFPL3
Exc THEMIS MBP
Exc RORB Epigenetic type 1
Exc RORB TSHZ2
Exc RORB PDE1C
Exc THEMIS SMYD1
Exc FEZF2 ZFHX3
Exc FEZF2 TRPM3
Exc FEZF2 NTNG1
Exc FEZF2 TRHDE
Exc FEZF2 PKD2L1
Inh TAFA1 GRIA4
Exc RORB QKI
Inh SST NPY
Inh VIP HTR3A
Inh TAFA1 TENM3
distance

Suppl. Fig. 6. Hierarchical clustering of the cell types based on ATAC-Seq. The 70 weighted nearest neighbor cell clusters were hierarchically clustered with L_1 norm (Manhattan distance), which is suggested as more suitable for high-dimension data, and with the average linkage agglomerative clustering algorithm using all detected ATAC features *(n=200 791)* and all samples *(n=79)* and cells *(n=180 016)* with TF-IDF-normalized counts. Icon art generated with BioRender.com (*Created in BioRender. Lee, J. (2026) https://BioRender.com/vb4wixj*).

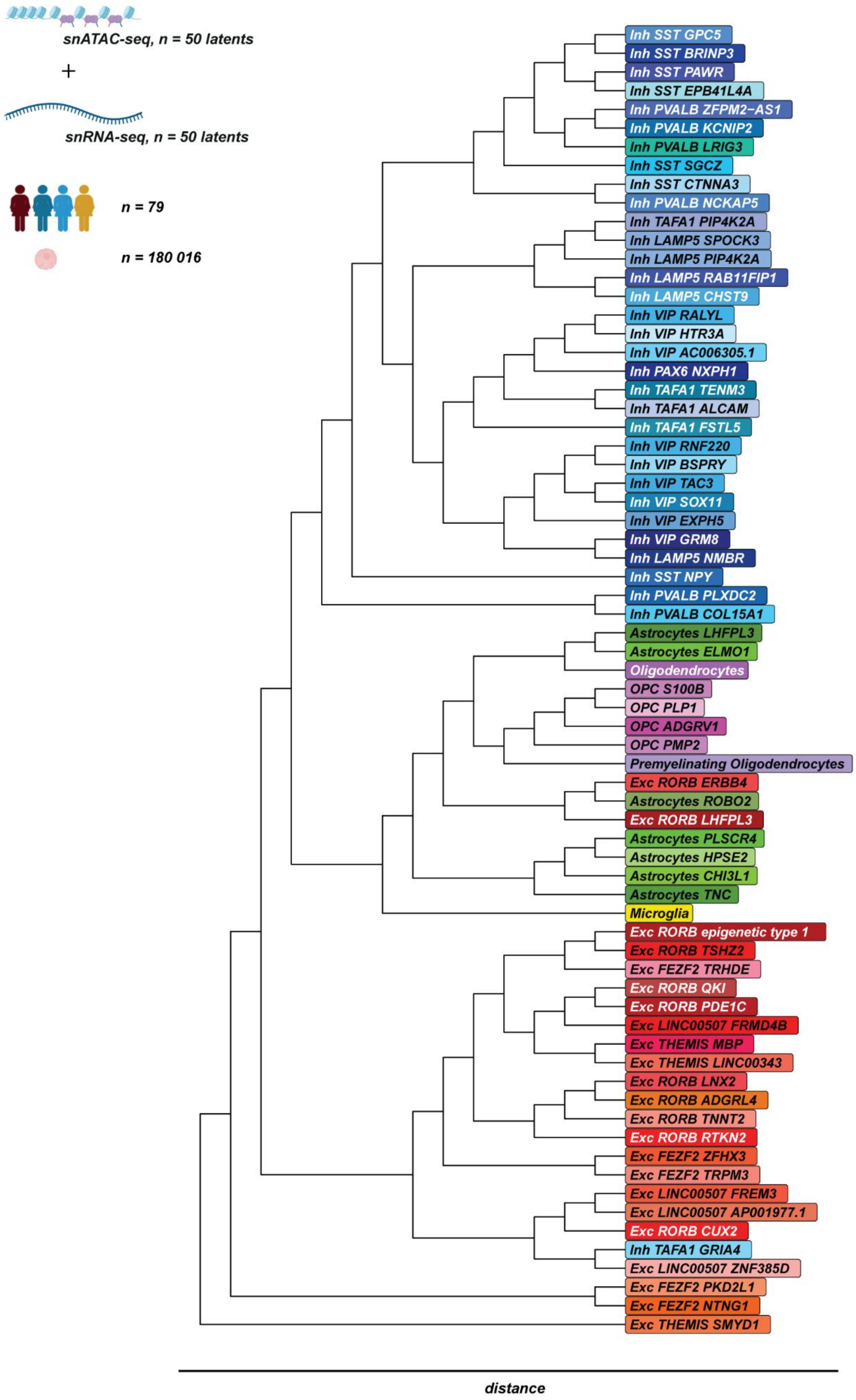

snATAC-seq, n = 50 latents
+
snRNA-seq, n = 50 latents
n = 79
n = 180 016
Inh SST GPC5
Inh SST BRINP3
Inh SST PAWR
Inh SST EPB41L4A
Inh PVALB ZFPM2−AS1
Inh PVALB KCNIP2
Inh PVALB LRIG3
Inh SST SGCZ
Inh SST CTNNA3
Inh PVALB NCKAP5
Inh TAFA1 PIP4K2A
Inh LAMP5 SPOCK3
Inh LAMP5 PIP4K2A
Inh LAMP5 RAB11FIP1
Inh LAMP5 CHST9
Inh VIP RALYL
Inh VIP HTR3A
Inh VIP AC006305.1
Inh PAX6 NXPH1
Inh TAFA1 TENM3
Inh TAFA1 ALCAM
Inh TAFA1 FSTL5
Inh VIP RNF220
Inh VIP BSPRY
Inh VIP TAC3
Inh VIP SOX11
Inh VIP EXPH5
Inh VIP GRM8
Inh LAMP5 NMBR
Inh SST NPY
Inh PVALB PLXDC2
Inh PVALB COL15A1
Astrocytes LHFPL3
Astrocytes ELMO1
Oligodendrocytes
OPC S100B
OPC PLP1
OPC ADGRV1
OPC PMP2
Premyelinating Oligodendrocytes
Exc RORB ERBB4
Astrocytes ROBO2
Exc RORB LHFPL3
Astrocytes PLSCR4
Astrocytes HPSE2
Astrocytes CHI3L1
Astrocytes TNC
Microglia
Exc RORB epigenetic type 1
Exc RORB TSHZ2
Exc FEZF2 TRHDE
Exc RORB QKI
Exc RORB PDE1C
Exc LINC00507 FRMD4B
Exc THEMIS MBP
Exc THEMIS LINC00343
Exc RORB LNX2
Exc RORB ADGRL4
Exc RORB TNNT2
Exc RORB RTKN2
Exc FEZF2 ZFHX3
Exc FEZF2 TRPM3
Exc LINC00507 FREM3
Exc LINC00507 AP001977.1
Exc RORB CUX2
Inh TAFA1 GRIA4
Exc LINC00507 ZNF385D
Exc FEZF2 PKD2L1
Exc FEZF2 NTNG1
Exc THEMIS SMYD1
distance

Suppl. Fig. 7 Hierarchical clustering of the cell types based on ATAC-Seq and transcriptome together. The 70 weighted nearest neighbor cell clusters were hierarchically clustered with L_1 norm (Manhattan distance), which is suggested as more suitable for high-dimension data, and with the average linkage agglomerative clustering algorithm using a reduced dimensionality space spanned by the 50 latents generated by scVI for the RNA-seq data and the 50 latents generated by peakVI for the ATAC-seq data and all samples *(n=79)* and cells *(n=180 016)* with log-normalized counts. Icon art generated with BioRender.com (Created in BioRender. Lee, J. (2026) https://BioRender.com/ovzweco).

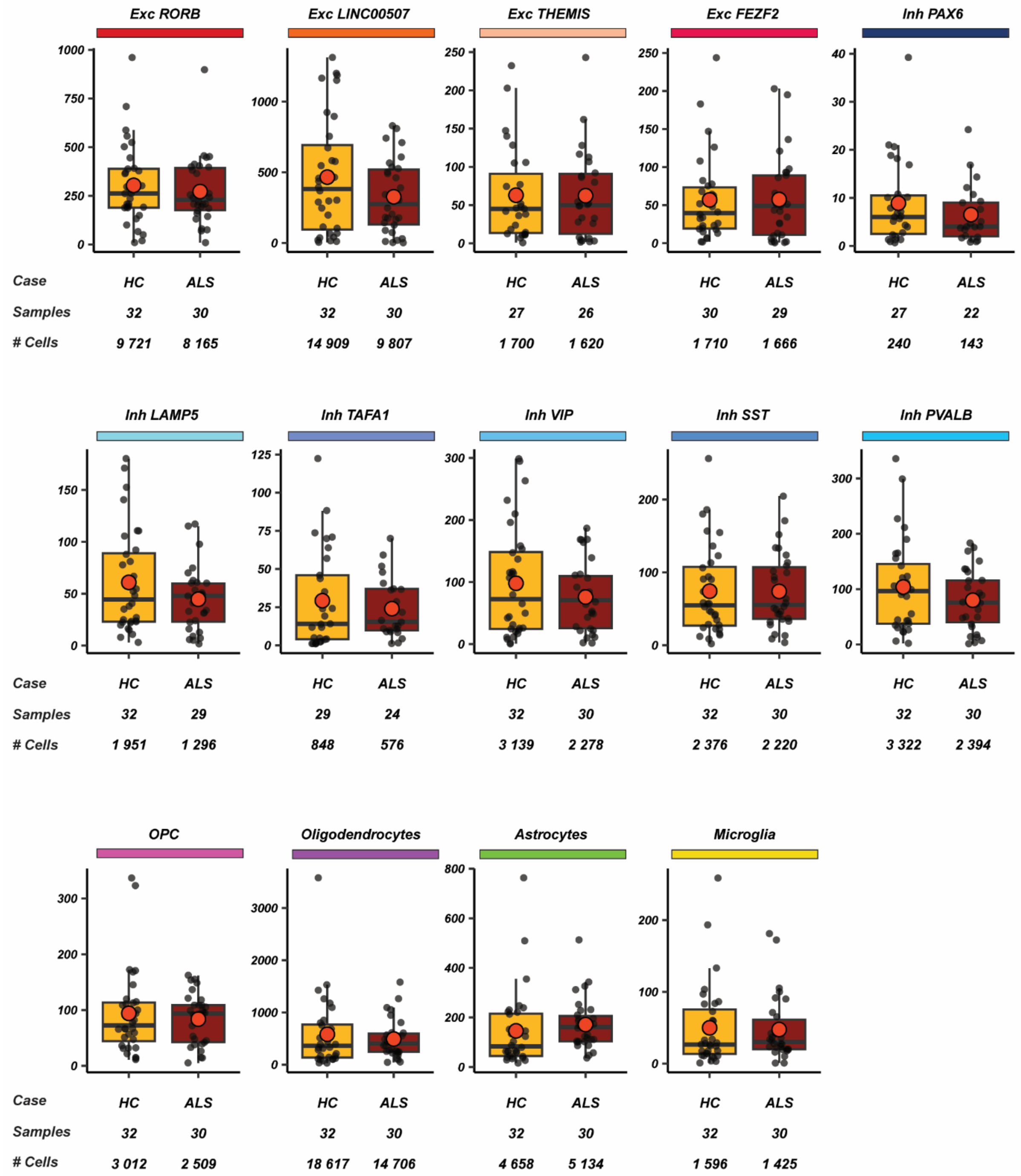

Suppl. Fig. 8 Number of nuclei per donor in each major cell type in the control and ALS group. Box plots with median (center line), first and third quartiles (25/75%), minimum/maximum value within 1.5x interquartile range from the first/third quantile, respective (lower/upper whisker), and mean average (orange points). Number of samples

(donors) from which the cell type was detected and total number of cells (nuclei) below graph.

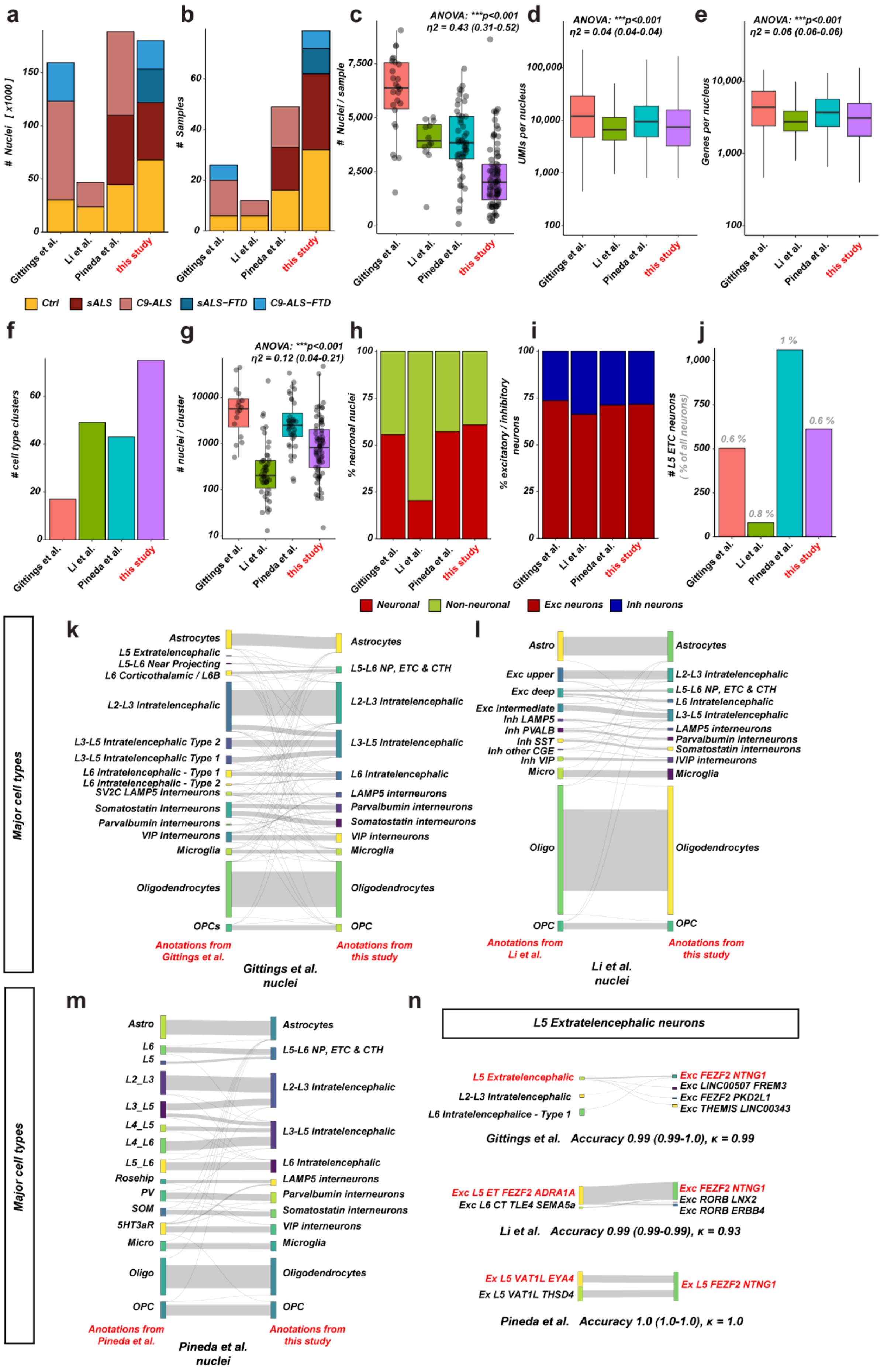
a
b
c
d
e
Nuclei [x1000]
Samples
Nuclei / sample
UMIs per nucleus
Genes per nucleus
ANOVA: ***p<0.001
η2 = 0.43 (0.31-0.52)
ANOVA: ***p<0.001
η2 = 0.04 (0.04-0.04)
ANOVA: ***p<0.001
η2 = 0.06 (0.06-0.06)
Gittings et al.
Li et al.
Pineda et al.
this study
Ctrl
sALS
C9-ALS
sALS−FTD
C9-ALS−FTD
f
g
h
i
j
cell type clusters
nuclei / cluster
ANOVA: ***p<0.001
η2 = 0.12 (0.04-0.21)
% neuronal nuclei
% excitatory / inhibitory neurons
# L5 ETC neurons
(% of all neurons)
0.6 %
0.8 %
1 %
0.6 %
Neuronal
Non-neuronal
Exc neurons
Inh neurons
k
l
Major cell types
Astrocytes
L5 Extratelencephalic
L5-L6 Near Projecting
L6 Corticothalamic / L6B
L2-L3 Intratelencephalic
L3-L5 Intratelencephalic Type 2
L3-L5 Intratelencephalic Type 1
L6 Intratelencephalic - Type 1
L6 Intratelencephalic - Type 2
SV2C LAMP5 Interneurons
Somatostatin Interneurons
Parvalbumin interneurons
VIP Interneurons
Microglia
Oligodendrocytes
OPCs
Astrocytes
L5-L6 NP, ETC & CTH
L2-L3 Intratelencephalic
L3-L5 Intratelencephalic
L6 Intratelencephalic
LAMP5 interneurons
Parvalbumin interneurons
Somatostatin interneurons
VIP interneurons
Microglia
Oligodendrocytes
OPC
Anotations from Gittings et al.
Anotations from this study
Gittings et al. nuclei
Astro
Exc upper
Exc deep
Exc intermediate
Inh LAMP5
Inh PVALB
Inh SST
Inh other CGE
Inh VIP
Micro
Oligo
OPC
Astrocytes
L2-L3 Intratelencephalic
L5-L6 NP, ETC & CTH
L6 Intratelencephalic
L3-L5 Intratelencephalic
LAMP5 interneurons
Parvalbumin interneurons
Somatostatin interneurons
IVIP interneurons
Microglia
Oligodendrocytes
OPC
Anotations from Li et al.
Anotations from this study
Li et al. nuclei
m
n
Astro
L6
L5
L2_L3
L3_L5
L4_L5
L4_L6
L5_L6
Rosehip
PV
SOM
5HT3aR
Micro
Oligo
OPC
Astrocytes
L5-L6 NP, ETC & CTH
L2-L3 Intratelencephalic
L3-L5 Intratelencephalic
L6 Intratelencephalic
LAMP5 interneurons
Parvalbumin interneurons
Somatostatin interneurons
VIP interneurons
Microglia
Oligodendrocytes
OPC
Anotations from Pineda et al.
Anotations from this study
Pineda et al. nuclei
L5 Extratelencephalic neurons
L5 Extratelencephalic
L2-L3 Intratelencephalic
L6 Intratelencephalice - Type 1
Exc FEZF2 NTNG1
Exc LINC00507 FREM3
Exc FEZF2 PKD2L1
Exc THEMIS LINC00343
Gittings et al. Accuracy 0.99 (0.99-1.0), κ = 0.99
Exc L5 ET FEZF2 ADRA1A
Exc L6 CT TLE4 SEMA5a
Exc FEZF2 NTNG1
Exc RORB LNX2
Exc RORB ERBB4
Li et al. Accuracy 0.99 (0.99-0.99), κ = 0.93
Ex L5 VAT1L EYA4
Ex L5 VAT1L THSD4
Ex L5 FEZF2 NTNG1
Pineda et al. Accuracy 1.0 (1.0-1.0), κ = 1.0

Suppl. Fig. 9. Comparison of the multi-omic single-nucleus dataset generated in this study to 3 previously published datasets. Statistics from each dataset. Only nuclei from the ALS/ALS-FTD motor cortex (but not FTD) were considered in each dataset (except Gittings et al, from the frontal cortex). Total number of nuclei (**a**), total number of donors (samples) (**b**), number of nuclei per sample (**c**), number of UMIs (reads) per nucleus (**d**), number of detected features (genes) per nucleus (**e**), total number of cell type clusters (**f**), number of nuclei per cluster (**g**), percentage of neuronal nuclei (**h**), of excitatory nuclei from all neuronal nuclei (**i**) and of L5 ETC neuronal nuclei as total number and as percentage from all neuronal nuclei (**j**). Scatter plots with jitter for each sample (c) or cluster (g) and boxplots without jitter (too many points) for cells as observations (d, e). Grey number above bars in **j** indicate the percent of all neuronal nuclei. Barplots in a, b, f & k represent only one value per bar. Box plots with median (center line), first and third quartiles (25/75%) and minimum/maximum value within 1.5x interquartile range from the first/third quantile, respective (lower/upper whisker). **k-m** Sankey plots demonstrate the concordant annotation of major cell types across all three datasets. **n** Sankey plots for the annotation in each of the three comparable datasets of the L5 ETC neurons (FEZF2 NTNG1 in this study). Only nuclei are shown from each dataset that were annotated as FEZF2 NTNG1 with label transfer from our multi-omic dataset. Accuracy with 95% CI and κ values.

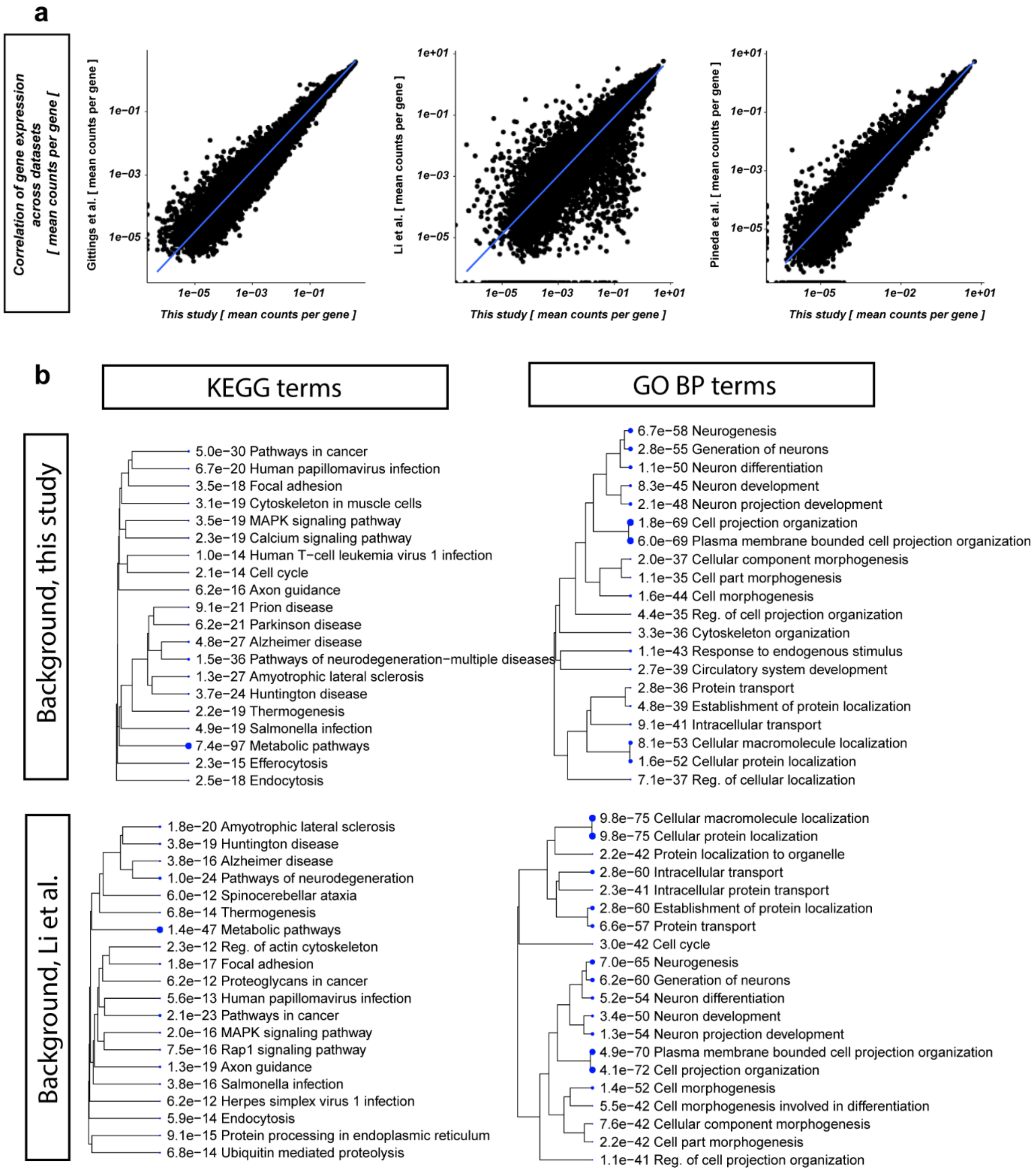

Suppl. Fig. 10. Comparable global gene expression in three similar single-nucleus RNA-seq datasets from the ALS/ALS-FTD motor cortex. a Correlation plots of the mean log1p-normalized expression for each gene detected in at least one of the both studies plotted. **B** KEGG term (left) and GO BP term (right) enrichment for all expressed genes in our dataset (top) and Li et al. (bottom) demonstrates the concordance of the genes that were detected in each study. For both studies, the background set is already enriched in pathways

related to neurodegenerative diseases and thus has to be considered in enrichment analyses. P-values adjusted with FDR.

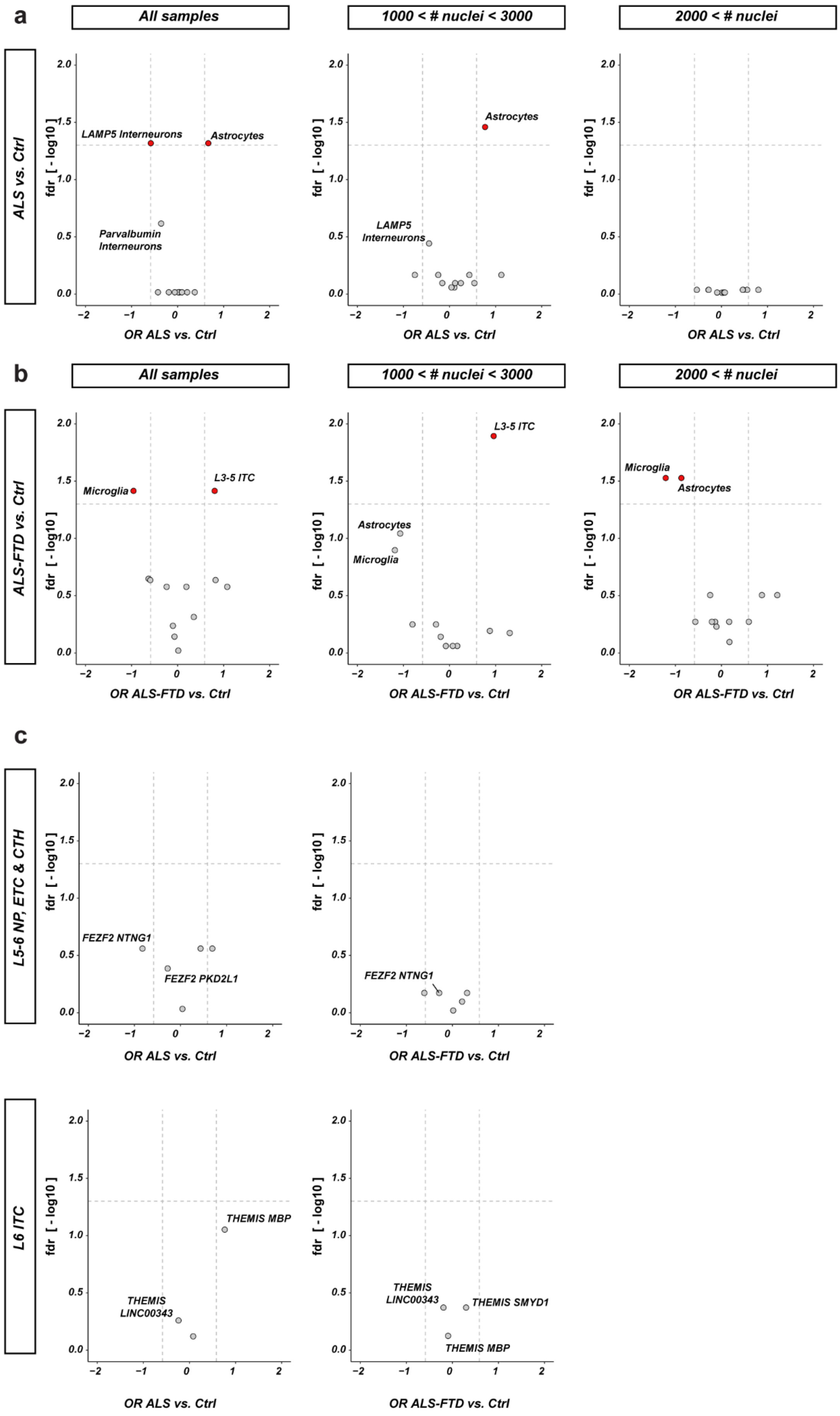

a
All samples
1000 < # nuclei < 3000
2000 < # nuclei
ALS vs. Ctrl
LAMP5 Interneurons
Astrocytes
Parvalbumin Interneurons
LAMP5 Interneurons
fdr [- log10]
OR ALS vs. Ctrl
b
ALS-FTD vs. Ctrl
Microglia
L3-5 ITC
Astrocytes
OR ALS-FTD vs. Ctrl
c
L5-6 NP, ETC & CTH
FEZF2 NTNG1
FEZF2 PKD2L1
L6 ITC
THEMIS MBP
THEMIS LINC00343
THEMIS SMYD1

Suppl. Fig. 11. Compositional cell-type analysis with mixed-effect linear models (MASC) in ALS and ALS-FTD. a, b MASC analysis with all samples (left), all samples with more than 1,000 and less than 3,000 nuclei (center) and with all samples with >2,000 nuclei in ALS (**a**) and ALS-FTD (**b**). fdr < 0.05 considered significant. X-axis: Odds ratio. **c** MASC analysis in L5 NP, ETC & CTH neurons (top) and L6 ITC neurons. No cell type was significantly altered, but a trend for decrease in the numbers of TDP-43 affected cell types, FEZF2 NTNG1 and THEMIS LINC00343 is visible. P-values adjusted with FDR.

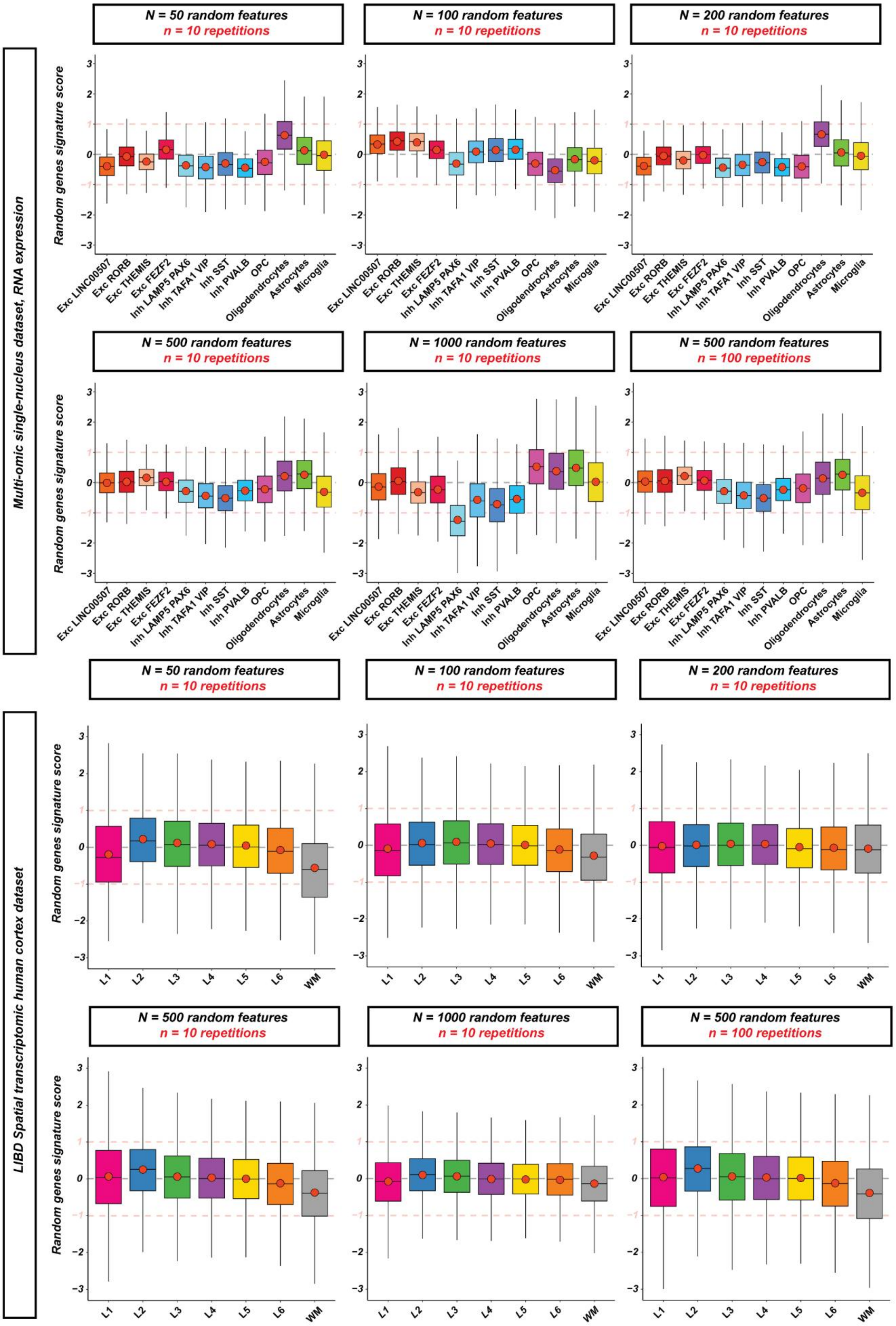
Multi-omic single-nucleus dataset, RNA expression
N = 50 random features
n = 10 repetitions
N = 100 random features
n = 10 repetitions
N = 200 random features
n = 10 repetitions
N = 500 random features
n = 10 repetitions
N = 1000 random features
n = 10 repetitions
N = 500 random features
n = 100 repetitions
Random genes signature score
3
2
1
0
-1
-2
-3
Exc LINC00507
Exc RORB
Exc THEMIS
Exc FEZF2
Inh LAMP5 PAX6
Inh TAFA1 VIP
Inh SST
Inh PVALB
OPC
Oligodendrocytes
Astrocytes
Microglia
LIBD Spatial transcriptomic human cortex dataset
N = 50 random features
n = 10 repetitions
N = 100 random features
n = 10 repetitions
N = 200 random features
n = 10 repetitions
N = 500 random features
n = 10 repetitions
N = 1000 random features
n = 10 repetitions
N = 500 random features
n = 100 repetitions
Random genes signature score
L1
L2
L3
L4
L5
L6
WM

Suppl.Fig. 12. Gene expression module accessibility score controls. Random sampling controls were performed as negative control for data analyses including the calculation of module scores for gene signatures. We performed 10 rounds of random controls with 50, 100, 200, 500 or 1000 randomly sampled features from the respective datasets, and 100 round with 500 randomly sampled features and summarized the module score per cell from the 10/100 rounds in the different major cell types. Box plots with median (center line), first and third quartiles (25/75%), minimum/maximum value within 1.5x interquartile range from the first/third quantile, respective (lower/upper whisker) and mean (red bullets). Dashed lines demonstrate the -1/1 threshold we observed for technical noise in the data.

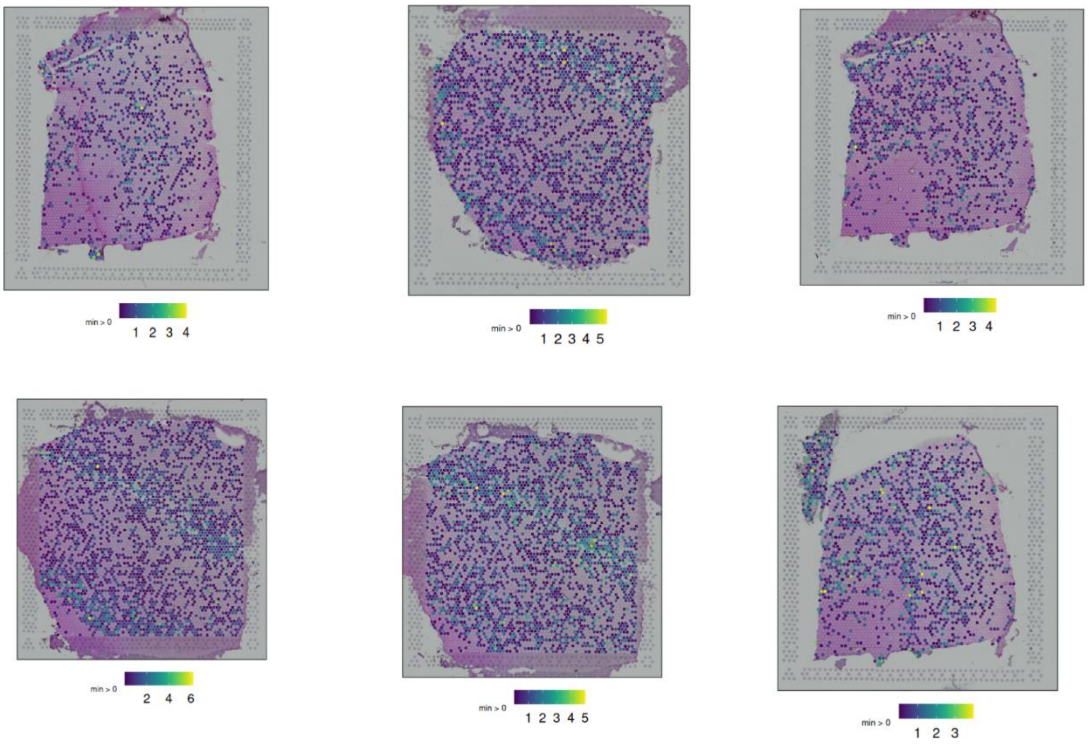

Suppl. Fig. 13. Extended visualization of L5 ETC localization (FEZF2 NTNG1 cell type) in the LIBD dataset. ModuleScore for FEZF2 NTNG markers were visualized in all slides as in Fig. 4. Note the different color scale for each slide.

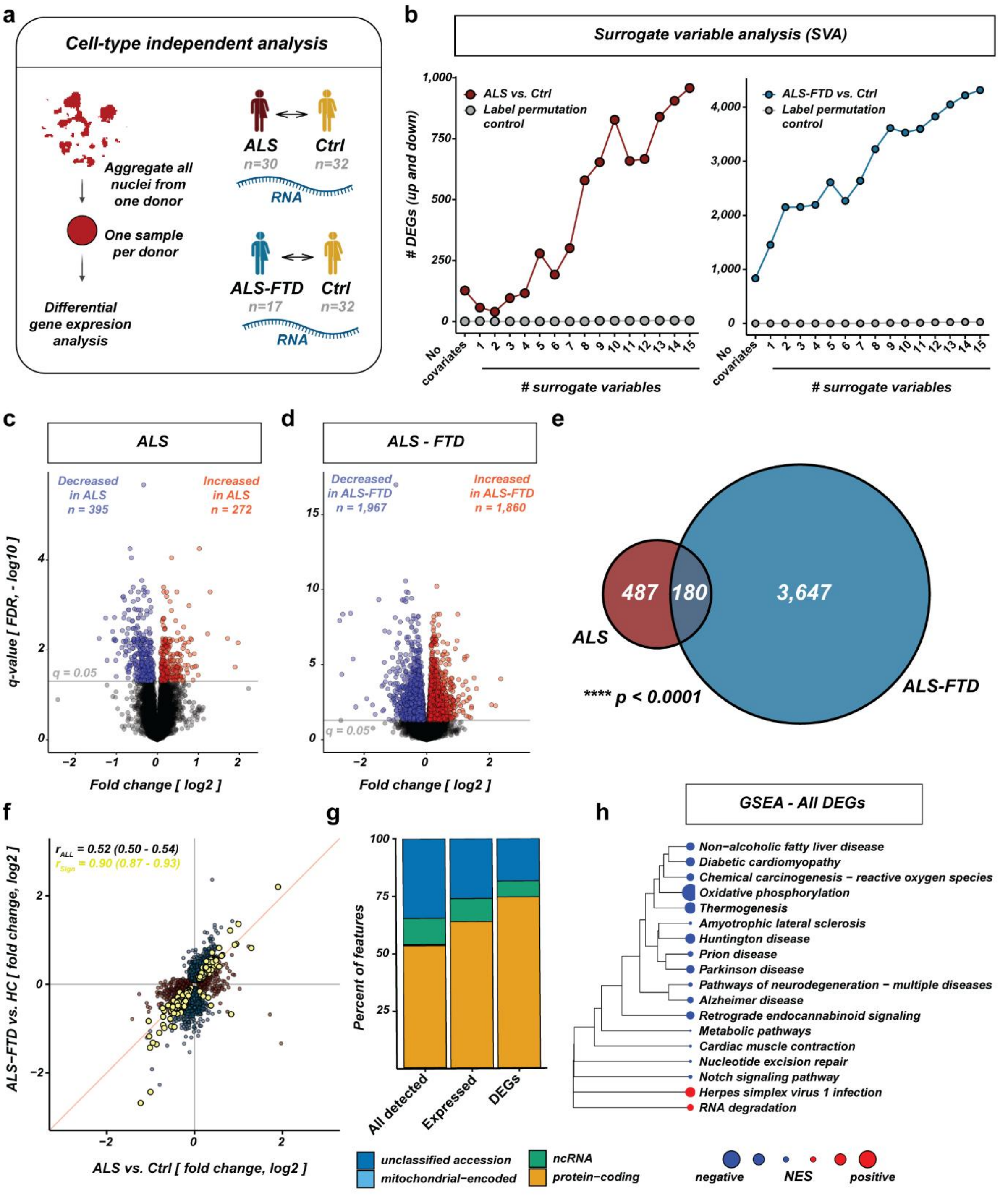

Suppl. Fig. 14. Cell-type independent ('pseudo-bulk') differential gene expression analysis. a Experimental setup. The gene counts for all nuclei per sample were pooled together to analyze differential gene expression irrespective of cell-type contributions. **b** Number of differentially expressed genes ('DEGs', $q < 0.05$, absolute log2 fold change > 0) vs. number of surrogate variables included as covariates. **c, d** Differentially expressed genes

in ALS and ALS-FTD (*q* < *0.05*, absolute log2 fold change > 0, 12 surrogate variables included as covariates). **e** Overlap of DEGs in ALS and ALS-FTD *(****p < 0.0001, Fisher's exact test, odds ratio: 2.1, 95% CI:1.8-2.5)*. A total of 4,314 unique DEGs were detected in both comparisons together. **f** Transcriptional changes across all 4,314 detected DEGs in ALS (x-axis) and ALS-FTD (y-axis). Most genes were concordantly changed in ALS and ALS-FTD, even if not statistically significant (yellow points: genes that were statistically significantly changed in both disease groups, blue points: significant only in the ALS-FTD comparison, red points: significant only in the ALS comparison; rALL = Pearson's correlation coefficient across all DEGs, rSign = Pearson's correlation coefficient across the 180 DEGs that were significant in both comparisons). **g** Classification of gene/transcript types. ~50 % of the detected features were protein-coding genes, ~33 % unclassified GeneBank accessions and ~ 11 % non-coding RNA ('ncRNA') genes. Genes that were expressed at least on the same levels as the DEG with the lowest expression ('Expressed') were enriched in protein-coding genes, and ALS/ALS-FTD DEGs even more. **h** Gene set enrichment analysis ('GSEA') of all 4,314 DEGs detected in ALS/ALS-FTD across all KEGG categories/terms. Terms were clustered hierarchically based on the size of the overlap of the core enrichment genes. Increasing dot size represents stronger positive (enrichment, red) or negative (decrease, blue normalized enrichment score ('NES') for the respective term. Threshold for considering a KEGG term significantly enriched: q < 0.05. All p-value adjustment were performed with FDR. Icon art generated with BioRender.com (Created in BioRender. Lee, J. (2026) https://BioRender.com/k8rcyv7).

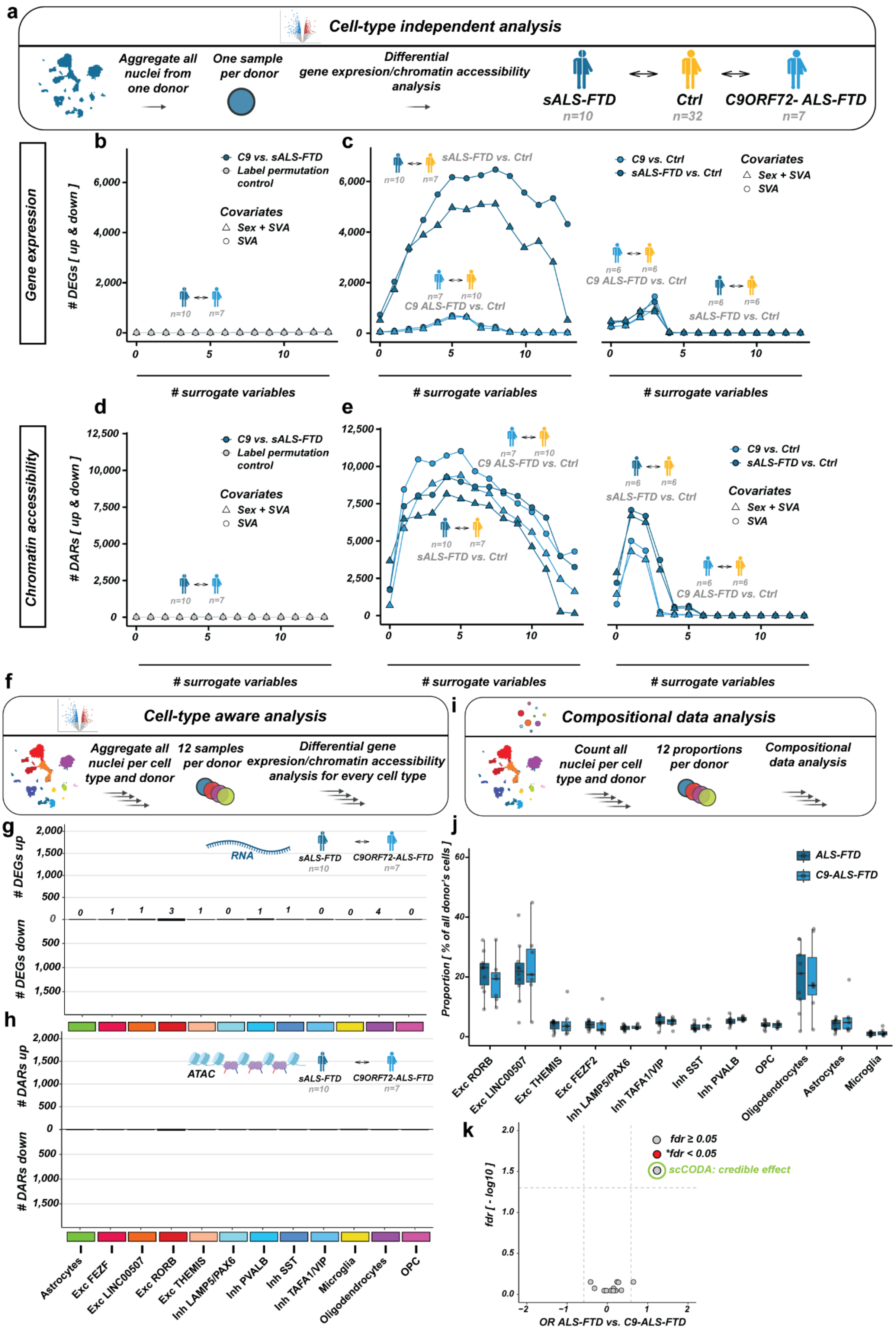

a
Cell-type independent analysis
Aggregate all nuclei from one donor
One sample per donor
Differential gene expression/chromatin accessibility analysis
sALS-FTD n=10
Ctrl n=32
C9ORF72- ALS-FTD n=7
Gene expression
b
DEGs [up & down]
C9 vs. sALS-FTD
Label permutation control
Covariates
Sex + SVA
SVA
surrogate variables
c
sALS-FTD vs. Ctrl
C9 ALS-FTD vs. Ctrl
C9 vs. Ctrl
sALS-FTD vs. Ctrl
Chromatin accessibility
d
DARs [up & down]
e
f
Cell-type aware analysis
Aggregate all nuclei per cell type and donor
12 samples per donor
Differential gene expression/chromatin accessibility analysis for every cell type
i
Compositional data analysis
Count all nuclei per cell type and donor
12 proportions per donor
Compositional data analysis
g
DEGs up
DEGs down
RNA
sALS-FTD
C9ORF72-ALS-FTD
h
DARs up
DARs down
ATAC
Astrocytes
Exc FEZF
Exc LINC00507
Exc RORB
Exc THEMIS
Inh LAMP5/PAX6
Inh PVALB
Inh SST
Inh TAFA1/VIP
Microglia
Oligodendrocytes
OPC
j
Proportion [% of all donor's cells]
ALS-FTD
C9-ALS-FTD
Exc FEZF2
Inh LAMP5/PAX6
k
fdr [- log10]
fdr ≥ 0.05
*fdr < 0.05
scCODA: credible effect
OR ALS-FTD vs. C9-ALS-FTD

Suppl. Fig. 15. Differential gene expression, chromatin accessibility and compositional data analyses in *C9ORF72* vs. sporadic ALS-FTD patients. a Schematic illustration of the analyses. Nuclei from one donor were pooled to a single sample ('pseudo-bulk') and sALS-FTD patients and *C9ORF72*-ALS-FTD patients compared to each other or to the neurologically unaffected controls ('Ctrl'). **b, c** Differential gene expression analysis. The number of differentially expressed genes ('DEGs') is plotted against the number of included surrogate variables ('SVs") from the SVA analysis as covariates in the DE analysis. Sex was also included as a covariate, as sample groups were not well balanced for sex. Negative control by a random permutation control. Comparison of *C9ORF72*-ALS-FTD to sALS-FTD did not detect significant transcriptomic differences between the two groups (**b**). Comparison of each disease group to the unaffected control group demonstrated more pronounced gene expression changes in sALS-FTD than in *C9ORF72*-ALS-FTD (**c**, left), but this difference was not observed when the two comparisons were equalized for statistical power (through same number of samples, **c**, right side). **d, e** Similarly to gene expression, chromatin accessibility was not significantly different between *C9ORF72*-ALS-FTD and sALS-FTD and numbers of detected DARs were similar when both disease groups were compared to the neurologically unaffected controls. **f-h** Differential gene expression (DE) and differential chromatin accessibility (DA) analysis in the 12 major cell types. No significant transcriptomic and epigenetic changes could be observed between *C9ORF72*-ALS-FTD and sporadic ALS-FTD. **i-k** Compositional data analysis with mixed linear models and scCODA did not detect any significant cell-type changes in the *C9ORF72*-ALS-FTD motor cortex when compared to sporadic ALS-FTD. **j** Percentages of major cell types in each group. Box plots with median (center line), first and third quartiles (25/75%) and minimum/maximum value within 1.5x interquartile range from the first/third quantile, respective (lower/upper whisker). Each dot is a sample donor. **k** Scatter plots of linear mixed models' odds ratio (OR) and fdr, significant results are marked with red points (mixed linear models, fdr < 0.05) and green circles (scCODA credible effect). Icon art generated with BioRender.com (Created in BioRender. Lee, J. (2026) https://BioRender.com/fuy816o).

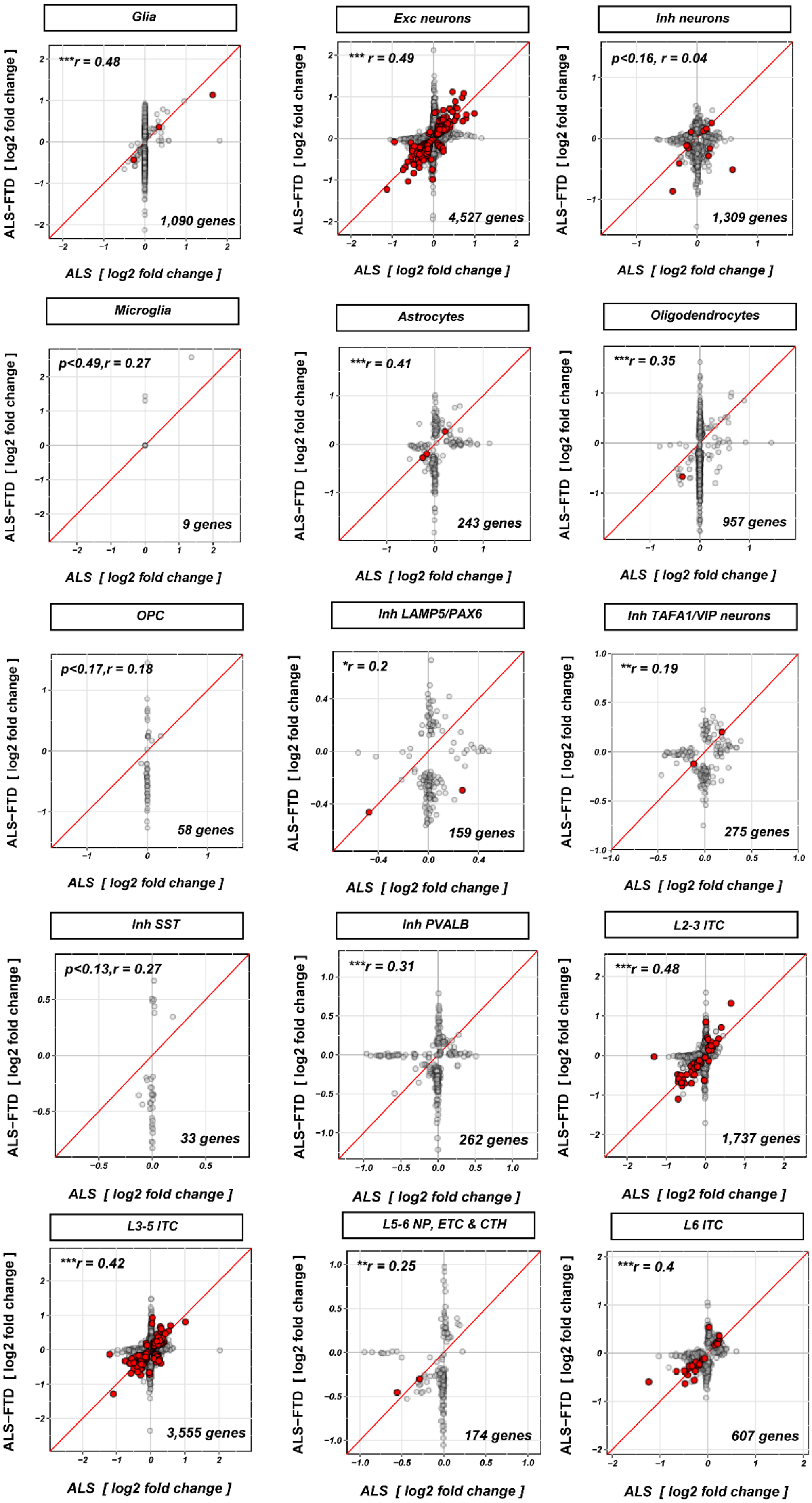
Glia
***r = 0.48
1,090 genes
Exc neurons
*** r = 0.49
4,527 genes
Inh neurons
p<0.16, r = 0.04
1,309 genes
Microglia
p<0.49,r = 0.27
9 genes
Astrocytes
***r = 0.41
243 genes
Oligodendrocytes
***r = 0.35
957 genes
OPC
p<0.17,r = 0.18
58 genes
Inh LAMP5/PAX6
*r = 0.2
159 genes
Inh TAFA1/VIP neurons
**r = 0.19
275 genes
Inh SST
p<0.13,r = 0.27
33 genes
Inh PVALB
***r = 0.31
262 genes
L2-3 ITC
***r = 0.48
1,737 genes
L3-5 ITC
***r = 0.42
3,555 genes
L5-6 NP, ETC & CTH
**r = 0.25
174 genes
L6 ITC
***r = 0.4
607 genes
ALS–FTD [log2 fold change]
ALS [log2 fold change]

Suppl. Fig. 16. Correlation of gene expression changes between ALS and ALS-FTD in each cell class and major cell type. Each scatter plot depicts the expression of all genes that were differentially expressed genes (DEGs) in the respective cell class/type in at least one of the comparisons ALS vs. Ctrl. Or ALS-FTD vs. Ctrl. Grey dots: genes which were DEG only in one of the comparisons. Red dots: DEGs that were significantly dysregulated in both disease groups. Pearson correlation on all genes (grey + red dots). *p<0.05, **p<0.01, ***p<0.001. Number of total genes in bottom right corner. Red line: identity line.

a

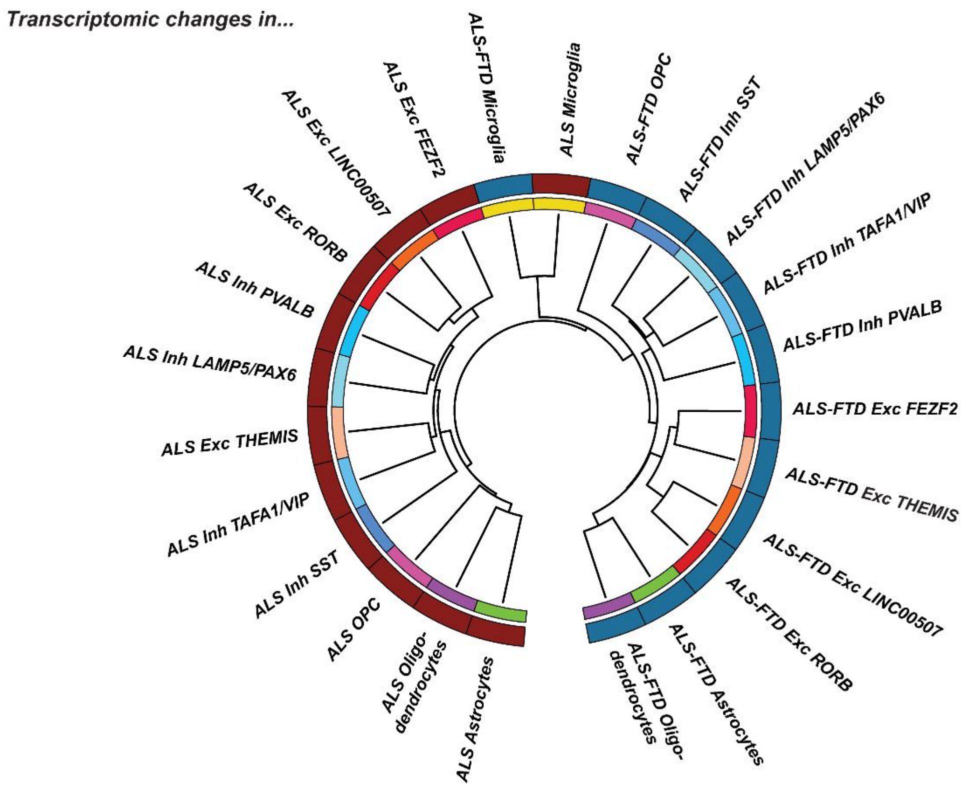
Transcriptomic changes in...
ALS-FTD Microglia
ALS Microglia
ALS-FTD OPC
ALS-FTD Inh SST
ALS-FTD Inh LAMP5/PAX6
ALS-FTD Inh TAFA1/VIP
ALS-FTD Inh PVALB
ALS-FTD Exc FEZF2
ALS-FTD Exc THEMIS
ALS-FTD Exc LINC00507
ALS-FTD Exc RORB
ALS-FTD Astrocytes
ALS-FTD Oligo-dendrocytes
ALS Astrocytes
ALS Oligo-dendrocytes
ALS OPC
ALS Inh SST
ALS Inh TAFA1/VIP
ALS Exc THEMIS
ALS Inh LAMP5/PAX6
ALS Inh PVALB
ALS Exc RORB
ALS Exc LINC00507
ALS Exc FEZF2

b

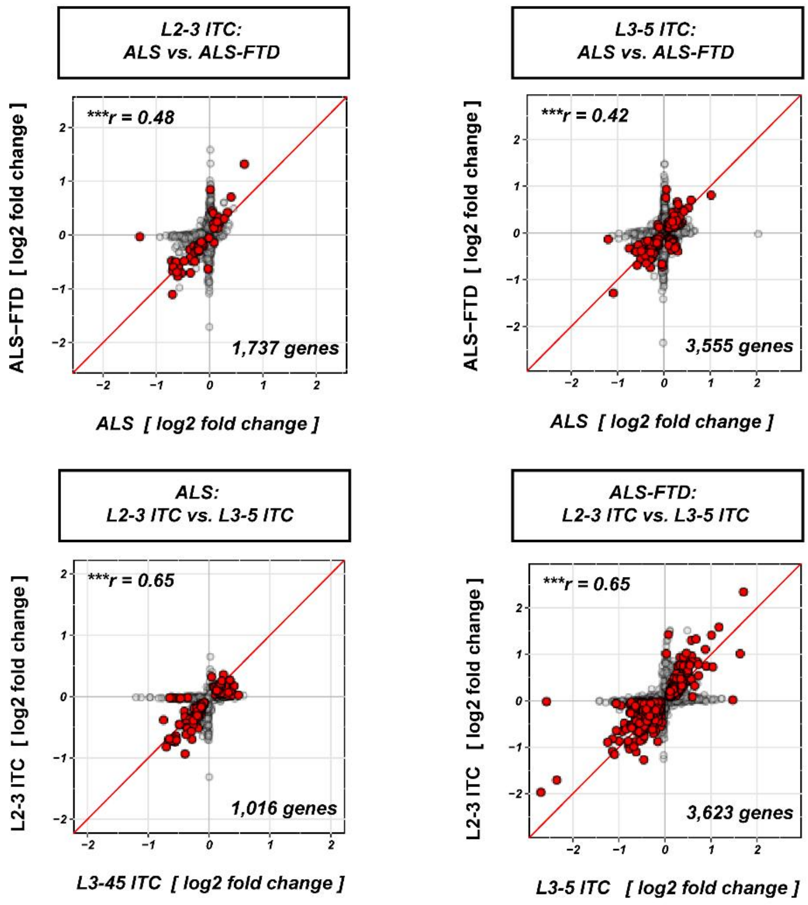
L2-3 ITC:
ALS vs. ALS-FTD
***r = 0.48
1,737 genes
ALS–FTD [log2 fold change]
ALS [log2 fold change]
L3-5 ITC:
ALS vs. ALS-FTD
***r = 0.42
3,555 genes
ALS–FTD [log2 fold change]
ALS [log2 fold change]
ALS:
L2-3 ITC vs. L3-5 ITC
***r = 0.65
1,016 genes
L2-3 ITC [log2 fold change]
L3-45 ITC [log2 fold change]
ALS-FTD:
L2-3 ITC vs. L3-5 ITC
***r = 0.65
3,623 genes
L2-3 ITC [log2 fold change]
L3-5 ITC [log2 fold change]

Suppl. Fig. 17. Association of gene expression changes in major cell types in ALS and ALS-FTD. a The major cell types were clustered hierarchically based on their dysregulation of the 5,471 genes in the ALS/ALS-FTD transcriptomic signature (average linkage with Euclidean distance, dendrogram in the center). **b** Correlation analysis demonstrated that the correlation of gene expression changes is stronger between different major cell types in the same disease group (bottom) than the correlation between different disease groups in the same major cell type (top). Each scatter plot depicts the expression of all genes that were differentially expressed genes (DEGs) in the respective comparisons labeled on the x and y axis. Grey dots: genes which were DEG only in one of the comparisons. Red dots: DEGs that were significantly dysregulated in both disease groups. Pearson correlation on all genes (grey + red dots). *$p<0.05$, **$p<0.01$, ***$p<0.001$. Number of total genes in bottom right corner. Red line: identity line.

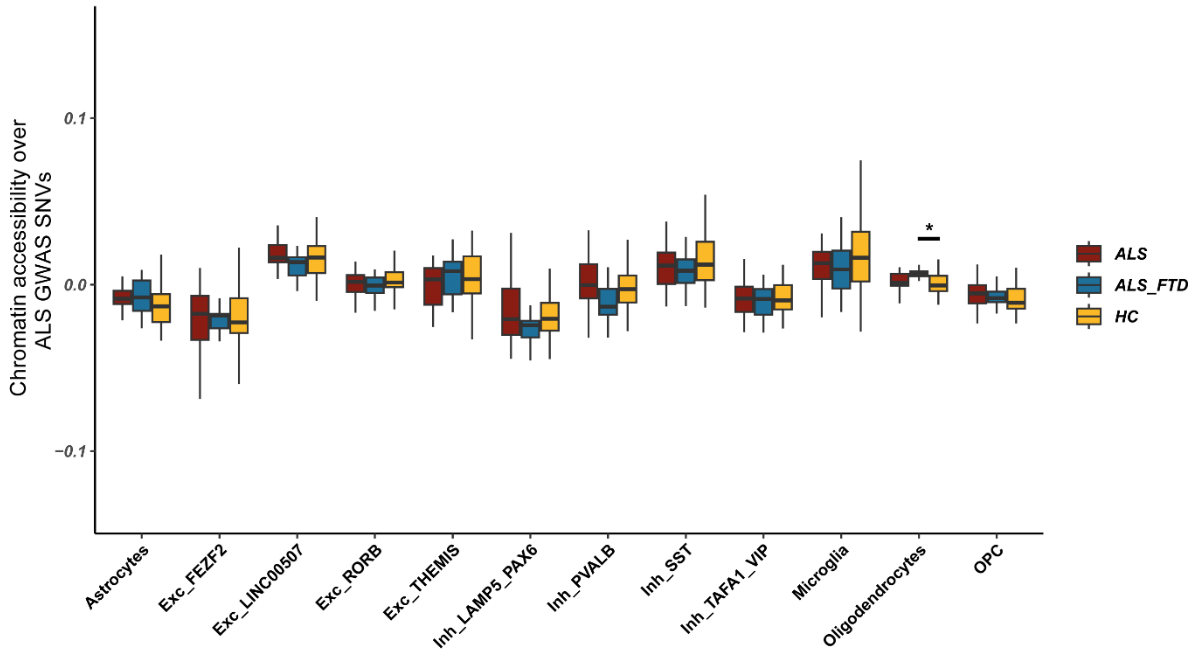

Suppl. Fig. 18. Chromatin accessibility over ALS GWAS SNVs. Combined chromatin accessibility ('module score') per cell type and case, summarized by individual. From a list of 222 unique, ALS-associated SNVs (GWASdb), 24 GWAS SNVs were overlapping a chromatin accessibility peak. Only ALS-FTD oligodendrocytes show a significant increase of chromatin accessibility over GWAS SNVs (Mann-Whitney U-test with fdr correction for multiple testing). *q<0.05. Box plots with median (center line), first and third quartiles (25/75%) and minimum/maximum value within 1.5x interquartile range from the first/third quantile, respective (lower/upper whisker).

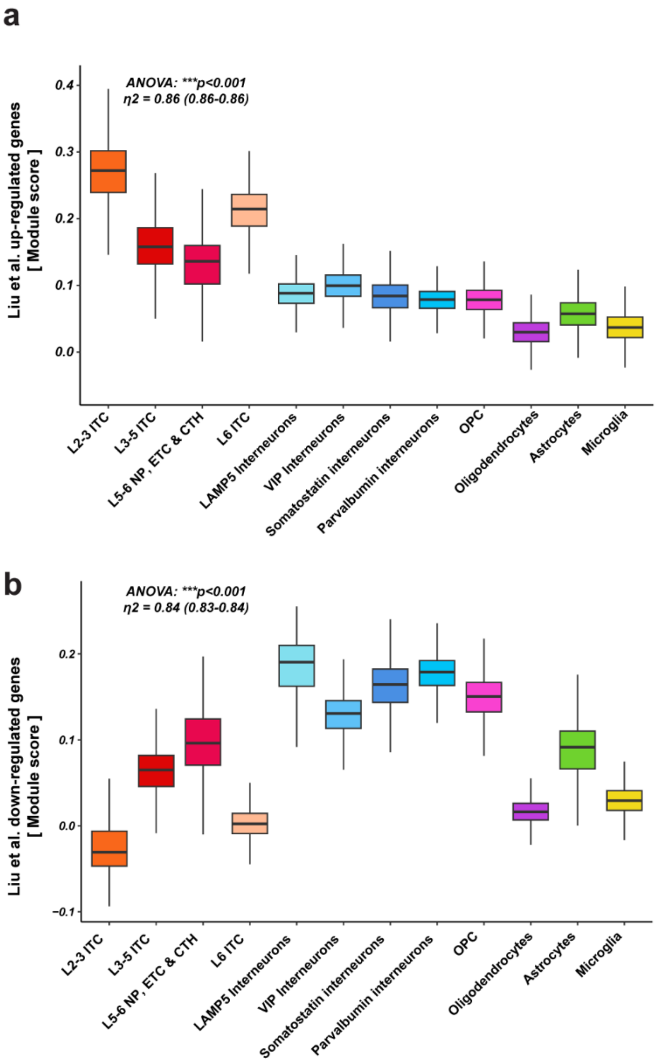

Suppl. Fig. 19. Enrichment of cell-type markers in differentially expressed genes in bulk-sequenced TDP-43 High vs TDP-43 Low nuclei from Liu et al. DEGs from differential gene expression analysis of the publicly available data from Liu et al. were used to build Module Scores for the up-regulated genes (in TDP-43 Low, upper panel) and the down-regulated genes (in TDP-43 Low, lower panel). ANOVA with eta squared for the effect of the major cell type, two-sided. Box plots with median (center line), first and third quartiles (25/75%) and minimum/maximum value within 1.5x interquartile range from the first/third quantile, respective (lower/upper whisker).

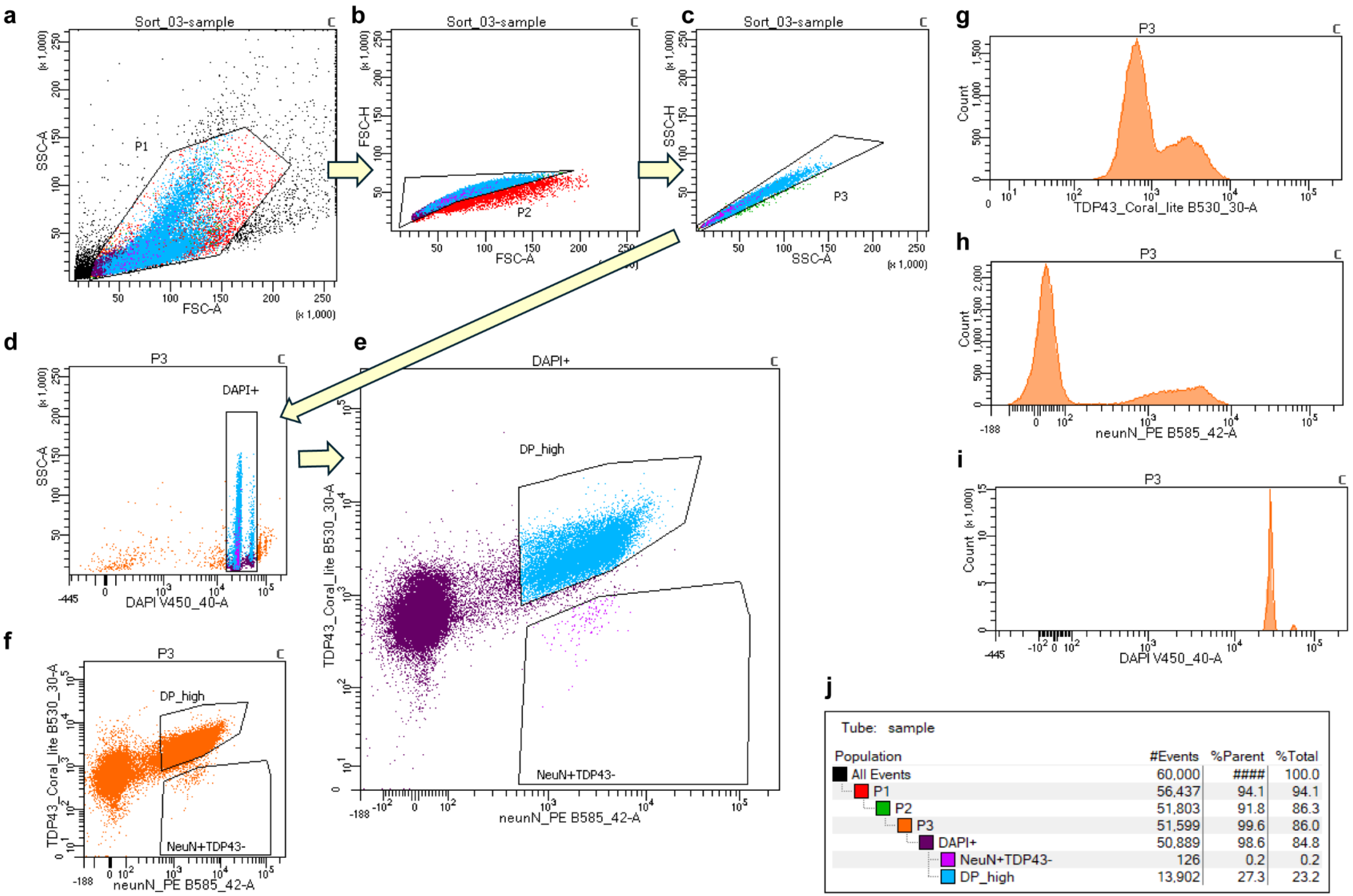

Population	#Events	%Parent	%Total
All Events	60,000	####	100.0
P1	56,437	94.1	94.1
P2	51,803	91.8	86.3
P3	51,599	99.6	86.0
DAPI+	50,889	98.6	84.8
NeuN+TDP43-	126	0.2	0.2
DP_high	13,902	27.3	23.2

Suppl. Fig. 20. Flow cytometry gating strategy for the fluorescence-activated nuclei sorting (FANS) coupled to sequencing. Nuclei were stained with DAPI (double-stranded DNA), NeuN-PE and TDP43-Coral and gated after compensation controls. First, nuclei were positively selected from debris based on sideward and forward scatter (**a**, with SSC-A and FSC-A, gate P1). Then, singlets were selected positively with FSC-A and FSC-H (**b**, gate P2) and SSC-A and SSC-H (**c**, gate P3). Then, stained and intact nuclei from smaller and larger cells were selected based on DAPI signal (two populations that either represent small and large nuclei or singlets and remaining doublets) (**d**). Please note that debris and damaged nuclei have much lower DAPI signal, while discretized signal higher than the selected may indicate multiplets. Typically, >80% of the nuclei were retained after this selection. Nuclei were than sorted into two channels, NeuN+ TDP-43 High ("DP_high" gate) and NeuN+ TDP43-Low ("NeuN+ TDP43-")(**e**). The gates were selected as to select TDP-43 Low cells relative to the NeuN signal (i.e. threshold for TDP-43 was lower for cells also with lower NeuN-signal), to ensure that nuclei with decreased TDP-43 signal just because of smaller size, damage or lower expression in the cell type are not erroneously selected. **f-i** Fluorescence signal intensity in gate P3 for all markers. **j** Representative count statistics for the sorting gates. The FANS-seq sorting experiment was performed n=4 times.

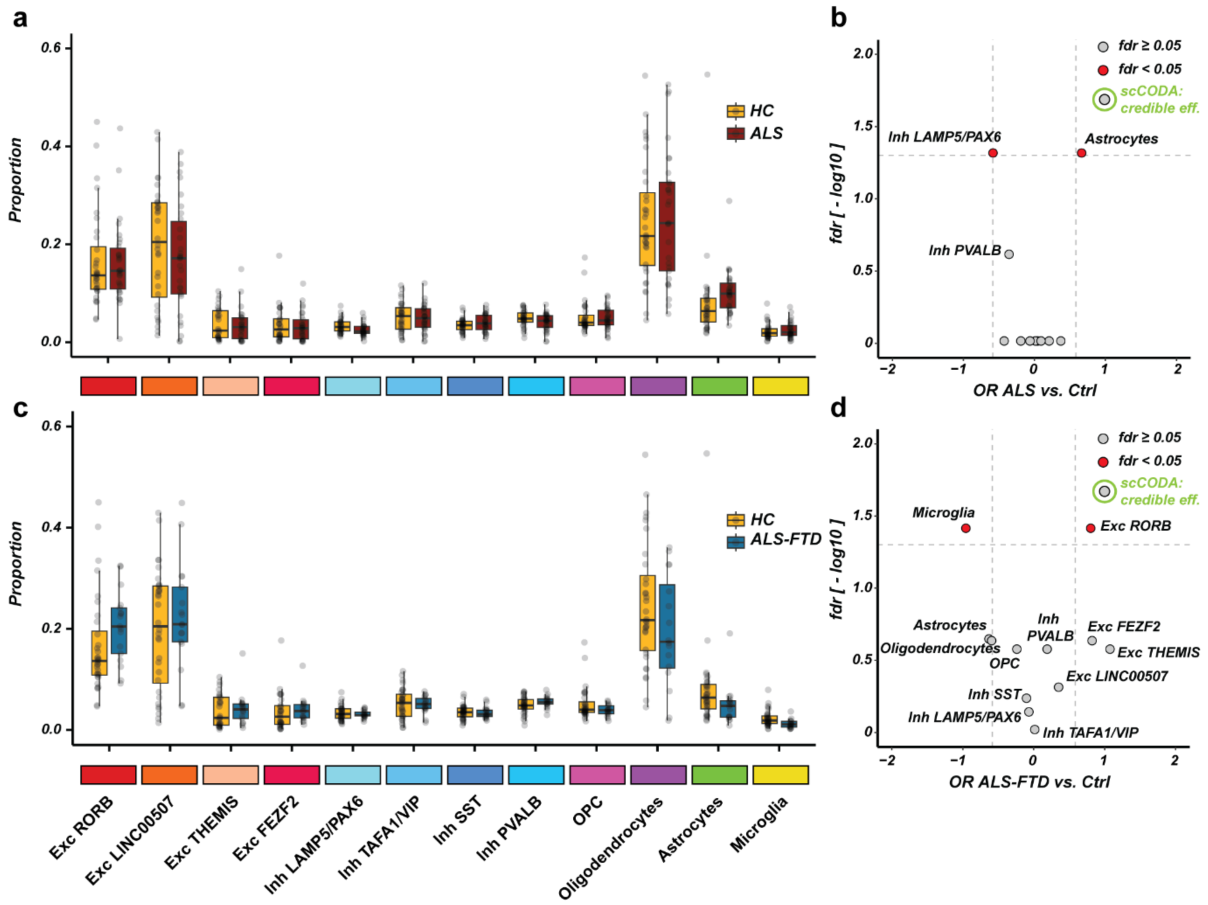

Suppl. Fig. 21. Compositional data analysis of the multi-omic single-nucleus motor cortex dataset. a, c Cell-type composition of the 12 major cell types in ALS (**a**) and ALS-FTD (**c**). Boxplots of the proportions of each major cell type (as percent of all cells of the respective donor) with median (center line), first and third quartiles (25/75%) and minimum/maximum value within 1.5x interquartile range from the first/third quantile, respective (lower/upper whisker). Each point is one sample donor. **b, d** Scatter plots of compositional data analysis of the 12 major cell types with mixed linear models in ALS (**b**) and ALS-FTD (**c**). We included sex and total number of cell from each donor as fixed effects and donor ID as a random effect. OR vs. fdr-corrected p-value. Significant results (fdr <

0.05) marked in red. Cell types for which the compositional data analysis with scCODA returned a significant/credible effect are marked with green circles (none in this analysis).
