# Supplementary material for: Multi-modal dissection of cell-type specific TDP-43 pathology in the motor cortex
Source: Nat Commun. 2026 Mar 9;17:2406. doi: 10.1038/s41467-026-69944-6 (PMC12982666; doi:10.1038/s41467-026-69944-6)
Supplement: Supplementary file 2 — Description of Additional Supplementary Files [file 41467_2026_69944_MOESM2_ESM.docx]

**Multi-modal dissection of cell-type specific TDP-43 pathology in the motor cortex**

Wolfgang P. Ruf, Julia K. Kühlwein, Laura Meier, Sarah J. Brockmann, Jaehyun LeeBae, Ghazaleh Sadri-Vakili, Deniz Yilmazer-Hanke, Susanne Petri, Dietmar R. Thal, Veselin Grozdanov, Karin M. Danzer

**Supplementary Data Legends**

**Supplementary Data 1. Summary of demographic characteristics of the disease/case groups for the multi-omic ALS-FTD motor cortex single-nuclei dataset.** Sample size: number of donors from which samples were included; age: mean ± standard deviation in years.

**Supplementary Data 2. Clinical and demographic characteristics of the disease and controls cases.** ID: Random anonymous identifier of each sample donor; age in years; C9ORF72: pathologic C9ORF72 hexanucleotide expansion; Genetic Variant: different polymorphisms detected; PMI: post-mortem interval in hours; age at onset in years; RIN: RNA-integrity number, measured before using the samples for single-nucleus sequencing. Cohort is not shown for each sample as a privacy measure.

**Supplementary Data 3. 10X Genomics Multiome Kit Chip & Well layout.** Samples were processed in different combinations on different wells of a 10X Genomics Multiome Kit chip as specified. Two to four samples’ nuclei were pooled during preparation, processed together on a well of the chip and demultiplexed in silico after sequencing based on sex (expression of genes on X and Y chromosomes, ATAC-seq peak reads on the X and Y chromosomes) and single-nucleotide polymorphisms (SNPs). 29/79 samples were processed on different 10X Genomics chip and wells on different experimental runs.

**Supplementary Data 4. Table of number of broad cell-type markers used for quantification of the gene signature scores in cells of the snRNA-seq data and spots of the LIBD spatial transcriptomics data.** "CellType": broad cell type, "n": number of gene expression markers identified with Wilcox test and passing the filtering (fdr and log2fc-threshold, both ENSEMBL and gene symbol identifiers available), "fdr": q-value (fdr) threshold applied, "log2fc": log2-fold change threshold applied.

**Supplementary Data 5. Table of markers of broad cell-types and extratelencephalic cells used for quantification of the gene signature scores in cells of the snRNA-seq data and spots of the LIBD spatial transcriptomics data.** Each cell type is listed on a separate xlsx-table sheet; 'ENSG': Ensembl gene identifier, 'SYMBOL': gene symbol.

**Supplementary Data 6. Table of numbers and percentages (of the whole dataset) across the hierarchical cell-type classification.** 'N': number of cells, 'Percentage': percentage of the whole dataset.

**Supplementary Data 7. Differential gene expression results from the cell-type unaware ('complete pseudo-bulk') comparison of ALS vs. Ctrl.** 'baseMean': average expression estimate as assessed by DESeq2; 'log2FoldChange': Log2 estimate of the expression fold change, negative values: decreased in ALS, positive values: increased in ALS; 'lfcSE': standard error estimate for the log2 fold change; 'stat': Wald's statistic; 'pvalue': p-value; 'padj': q-value (fdr-adjusted p-value); 'SYMBOL': HGNC gene symbol (or GenBank accession); all tests two-sided.

**Supplementary Data 8. Differential gene expression results from the cell-type unaware ('complete pseudo-bulk') comparison of ALS-FTD vs. Ctrl.** 'baseMean': average expression estimate as assessed by DESeq2; 'log2FoldChange': Log2 estimate of the expression fold change, negative values: decreased in ALS, positive values: increased in ALS; 'lfcSE': standard error estimate for the log2 fold change; 'stat': Wald's statistic; 'pvalue': p-value; 'padj': q-value (fdr-adjusted p-value); 'SYMBOL': HGNC gene symbol (or GenBank accession); all tests two-sided.

**Supplementary Data 9. Differentially expressed genes from the cell-type unaware ('complete pseudo-bulk') comparison of ALS vs. Ctrl and ALS-FTD vs. Ctrl.** All unique genes from both analyses listed. 'ID_CellRanger': Gene ID assigned by CellRanger from the 10X Genomics Human GRCh38 (GENCODE v32/Ensembl98) '2020-A' genomic reference; 'SYMBOL': HGNC gene symbol (or GenBank accession id if no HGNC symbol assigned); GENETYPE: annotated gene type; 'DEG_In': comparison in which the gene was significantly differentially expressed (q < 0.05).

**Supplementary Data 10. ALS/ALS-FTD DGE signature: 5,471 genes significant in at least one major cell type in ALS or at least one major cell type in ALS-FTD.** 'Gene': Gene Symbol; 'ID_10X': Gene Id in 10X Genomics GRCh38 2020 A Reference; 'GENETYPE': gene classification; 'Signif_In': Significant in ALS vs. Ctrl. only ('ALS'), ALS-FTD vs. Ctrl. only ('ALSFTD') or ALS vs. Ctrl. & ALS-FTD vs. Ctrl. ('Both').

**Supplementary Data 11. ALS/ALS-FTD DGE signature: 510 genes significant in at least one major cell type in ALS and at least one major cell type in ALS-FTD.** 'Gene': Gene Symbol; 'ID_10X': Gene Id in 10X Genomics GRCh38 2020 A Reference; 'GENETYPE': gene classification; 'Signif_In': Significant in ALS vs. Ctrl. only ('ALS'), ALS-FTD vs. Ctrl. only ('ALSFTD') or ALS vs. Ctrl. & ALS-FTD vs. Ctrl. ('Both').

**Supplementary Data 12. DGE results in pooled all nuclei pseudo-bulk per donor, TDP-43 High vs. TPD-43 Low.** 'baseMean': average expression estimate as assessed by DESeq2; 'log2FoldChange': Log2 estimate of the expression fold change, negative values: decreased in TDP-43 Low, positive values: increased in TDP-43 Low; 'lfcSE': standard error estimate for the log2 fold change; 'stat': Wald's statistic; 'pvalue': p-value; 'padj': q-value (fdr-adjusted p-value); 'SYMBOL': HGNC gene symbol (or ENSEMBL/Genbank accession); all tests two-sided.

**Supplementary Data 13. DGE results in Exc L5-6 NP, ETC & CTH FEZF2 nuclei TDP-43 High vs. TPD-43 Low.** 'baseMean': average expression estimate as assessed by DESeq2; 'log2FoldChange': Log2 estimate of the expression fold change, negative values: decreased in TDP-43 Low, positive values: increased in TDP-43 Low; 'lfcSE': standard error estimate for the log2 fold change; 'stat': Wald's statistic; 'pvalue': p-value; 'padj': q-value (fdr-adjusted p-value); 'SYMBOL': HGNC gene symbol (or ENSEMBL/Genbank accession); all tests two-sided.

**Supplementary Data 14. DGE results in Exc L3-5 ITC RORB nuclei TDP-43 High vs. TPD-43 Low.** 'baseMean': average expression estimate as assessed by DESeq2; 'log2FoldChange': Log2 estimate of the expression fold change, negative values: decreased in TDP-43 Low, positive values: increased in TDP-43 Low; 'lfcSE': standard error estimate for the log2 fold change; 'stat': Wald's statistic; 'pvalue': p-value; 'padj': q-value (fdr-adjusted p-value); 'SYMBOL': HGNC gene symbol (or ENSEMBL/Genbank accession); all tests two-sided.

**Supplementary Data 15. DGE results in Exc L2-3 ITC LINC00507 nuclei TDP-43 High vs. TPD-43 Low.** 'baseMean': average expression estimate as assessed by DESeq2; 'log2FoldChange': Log2 estimate of the expression fold change, negative values: decreased in TDP-43 Low, positive values: increased in TDP-43 Low; 'lfcSE': standard error estimate for the log2 fold change; 'stat': Wald's statistic; 'pvalue': p-value; 'padj': q-value (fdr-adjusted p-value); 'SYMBOL': HGNC gene symbol (or ENSEMBL/Genbank accession); all tests two-sided.

**Supplementary Data 16. DGE results in Exc L6 ITC THEMIS nuclei TDP-43 High vs. TPD-43 Low.** 'baseMean': average expression estimate as assessed by DESeq2; 'log2FoldChange': Log2 estimate of the expression fold change, negative values: decreased in TDP-43 Low, positive values: increased in TDP-43 Low; 'lfcSE': standard error estimate for the log2 fold change; 'stat': Wald's statistic; 'pvalue': p-value; 'padj': q-value (fdr-adjusted p-value); 'SYMBOL': HGNC gene symbol (or ENSEMBL/Genbank accession); all tests two-sided.

**Supplementary Data 17. DGE results in Exc FEZF2 NTNG1 nuclei TDP-43 High vs. TPD-43 Low.** 'gene': HGNC gene symbol (or ENSEMBL/Genbank accession); 'p_value': p-value; 'model_log2FC': model log2 fold-change; 'ci.hi': upper log2 fold-change 95% confidence estimate; 'ci.lo': lower log2 fold-change 95% confidence estimate; 'fdr': q-value(fdr-adjusted p-value); all tests two-sided.

**Supplementary Data 18. DGE results in Exc LINC00507 FREM3 nuclei TDP-43 High vs. TPD-43 Low.** 'gene': HGNC gene symbol (or ENSEMBL/Genbank accession); 'p_value': p-value; 'model_log2FC': model log2 fold-change; 'ci.hi': upper log2 fold-change 95% confidence estimate; 'ci.lo': lower log2 fold-change 95% confidence estimate; 'fdr': q-value (fdr-adjusted p-value); all tests two-sided.

**Supplementary Data 19. DGE results in Exc RORB ADGRL4 nuclei TDP-43 High vs. TPD-43 Low.** 'gene': HGNC gene symbol (or ENSEMBL/Genbank accession); 'p_value': p-value; 'model_log2FC': model log2 fold-change; 'ci.hi': upper log2 fold-change 95% confidence estimate; 'ci.lo': lower log2 fold-change 95% confidence estimate; 'fdr': q-value (fdr-adjusted p-value); all tests two-sided.

**Supplementary Data 20. DGE results in Exc RORB LNX2 nuclei TDP-43 High vs. TPD-43 Low.** 'gene': HGNC gene symbol (or ENSEMBL/Genbank accession); 'p_value': p-value; 'model_log2FC': model log2 fold-change; 'ci.hi': upper log2 fold-change 95% confidence estimate; 'ci.lo': lower log2 fold-change 95% confidence estimate; 'fdr': q-value (fdr-adjusted p-value); all tests two-sided.

**Supplementary Data 21. DGE results in Exc THEMIS LINC00343 nuclei TDP-43 High vs. TPD-43 Low.** 'gene': HGNC gene symbol (or ENSEMBL/Genbank accession); 'p_value': p-value; 'model_log2FC': model log2 fold-change; 'ci.hi': upper log2 fold-change 95% confidence estimate; 'ci.lo': lower log2 fold-change 95% confidence estimate; 'fdr': q-value (fdr-adjusted p-value); all tests two-sided.

**Supplementary Data 22. 'Per cell' statistics of the multi-omic dataset per donor.**

**Supplementary Data 23. Multi-omic dataset statistics per donor and major cell type.**

**Supplementary Data 24. Multi-omic dataset statistics per donor.**

**Supplementary Data 25. FANS-Seq Sample Info.**
