# Supplementary material for: Multi-modal dissection of cell-type specific TDP-43 pathology in the motor cortex
Source: Nat Commun. 2026 Mar 9;17:2406. doi: 10.1038/s41467-026-69944-6 (PMC12982666; doi:10.1038/s41467-026-69944-6)
Supplement: Supplementary file 3 — Supplementary Data 1-25 [file 41467_2026_69944_MOESM3_ESM.zip › Supplementary_Data_1.docx]

|  | **ALS** | **ALS-FTD** | **ALS-FTD-*C9ORF72*** | **Ctrl** |
| --- | --- | --- | --- | --- |
| ***Sample size*** | 30 | 10 | 7 | 32 |
| ***Sex [f/m] (%)*** | 9/21  (30/70 %) | 3/7  (30/70 %) | 4/3  (57/43 %) | 15/17 (47/53 %) |
| ***Age [years]*** | 61.2 ± 12.3 | 67.5 ± 8.4 | 62.7 ± 5.2 | 71.8 ± 18.5 |

**Supplementary Data 1. Summary of demographic characteristics of the disease/case groups for the multi-omic ALS-FTD motor cortex single-nuclei dataset.** Sample size: number of donors from which samples were included; age: mean ± standard deviation in years.
