# Supplementary material for: Multi-modal dissection of cell-type specific TDP-43 pathology in the motor cortex
Source: Nat Commun. 2026 Mar 9;17:2406. doi: 10.1038/s41467-026-69944-6 (PMC12982666; doi:10.1038/s41467-026-69944-6)
Supplement: Supplementary file 4 — Reporting Summary [file 41467_2026_69944_MOESM4_ESM.pdf]

nature portfolio

Corresponding author(s): Prof. Dr. Karin M. Danzer
Veselin Grozdanov, PhD

Last updated by author(s): Jan 30, 2026

Reporting Summary

Nature Portfolio wishes to improve the reproducibility of the work that we publish. This form provides structure for consistency and transparency in reporting. For further information on Nature Portfolio policies, see our Editorial Policies and the Editorial Policy Checklist.

Statistics

For all statistical analyses, confirm that the following items are present in the figure legend, table legend, main text, or Methods section.

n/a	Confirmed	
☐	☒	The exact sample size (*n*) for each experimental group/condition, given as a discrete number and unit of measurement
☐	☒	A statement on whether measurements were taken from distinct samples or whether the same sample was measured repeatedly
☐	☒	The statistical test(s) used AND whether they are one- or two-sided<br>*Only common tests should be described solely by name; describe more complex techniques in the Methods section.*
☐	☒	A description of all covariates tested
☐	☒	A description of any assumptions or corrections, such as tests of normality and adjustment for multiple comparisons
☐	☒	A full description of the statistical parameters including central tendency (e.g. means) or other basic estimates (e.g. regression coefficient) AND variation (e.g. standard deviation) or associated estimates of uncertainty (e.g. confidence intervals)
☐	☒	For null hypothesis testing, the test statistic (e.g. *F*, *t*, *r*) with confidence intervals, effect sizes, degrees of freedom and *P* value noted<br>*Give P values as exact values whenever suitable.*
☐	☒	For Bayesian analysis, information on the choice of priors and Markov chain Monte Carlo settings
☐	☒	For hierarchical and complex designs, identification of the appropriate level for tests and full reporting of outcomes
☐	☒	Estimates of effect sizes (e.g. Cohen's *d*, Pearson's *r*), indicating how they were calculated

Our web collection on statistics for biologists contains articles on many of the points above.

Software and code

Policy information about availability of computer code

Data collection	The custom code used in this study is available on GitHub under https://github.com/DanzerLab/Ruf_et_al_2026.
Data analysis	List of SW used:<br>10X Genomics cellranger count v9.0.1<br>10 Genomics cellranger arc 2.0.2<br>Souporcell v2.5<br>FACSDiva v9.0.1<br>SoupX<br>CellBender v0.3.0<br>PyTables v3.10.2<br>scDblFinder v1.20.2<br>Seurat v4.4.0 & v.5.0.1<br>MACS2 v2.2.6<br>Signac v1.10.0<br>scVI v1.0.0<br>scikit v1.8.0<br>PeakVI v1.2<br>Harmony v1.2.4<br>Bonsai-Scout

scCODA v0.1.9
sva v3.58.0
DESeq2 v1.50.0
BulkR v0.0.0.9001
seqtk v1.5
Ens.db.Hs.86 v2.99.0
org.Hs.eg.db v3.22.0
ShinyGO v0.85
StringDB v12.0
fgsea v1.36.0
clusterProfiler v4.18.1
tidyverse v2.0.0
Pandas v3.0.0
BioConductor v3.22
leafcutter
regtools v1.0.0
spatialLIBD v1.22.0
STARv2.7.11b
featureCounts v1.22.2
ChIPseeker v1.46.1
effsize v0.8.1
effectsize v1.0.1
caret v7.0.1
ggplot2 v4.0.0
complexHeatmap v2.26.0
apeglm v1.32.0
pheatmap v1.0.13
ggrastr v1.0.2
complexUpset v1.3.3
ggnewscale v0.5.2
patchwork v1.3.2
scattermore v1.2
ggraph v2.2.2
data.tree v1.2.0
igraph v2.2.1
networkD3 v0.4.1
ggrepel v0.9.6
ggpubr v0.6.1
dendextend v1.19.1
IGV browser
Further information can be found on the repository: https://github.com/DanzerLab/Ruf_et_al_2026.

For manuscripts utilizing custom algorithms or software that are central to the research but not yet described in published literature, software must be made available to editors and reviewers. We strongly encourage code deposition in a community repository (e.g. GitHub). See the Nature Portfolio guidelines for submitting code & software for further information.

Data

Policy information about availability of data

All manuscripts must include a data availability statement. This statement should provide the following information, where applicable:

- Accession codes, unique identifiers, or web links for publicly available datasets
- A description of any restrictions on data availability
- For clinical datasets or third party data, please ensure that the statement adheres to our policy

The raw sequencing data that was generated in this study is available on EGA under EGAD50000002240 (https://ega-archive.org/studies/EGAS50000001562) (multi-omic dataset) and EGAD50000002243 (https://ega-archive.org/datasets/EGAD50000002243) (FANS-seq dataset) and is subject to controlled access due to data privacy regulations (identifiable genomic sequence data in combination with phenotype). Requests for accessing the raw data are to be addressed to the Data Access Committee (EGAC50000000856) (https://ega-archive.org/dacs/EGAC50000000856) through the EGA platform. Requests will be reviewed by the Data Access Committee (expected timeframe: 25 working days), approved for use (in five years timeframe) if the application requires access to the raw genomic sequences and require the signing of a data transfer agreement to guarantee compliance with the European General Data Protection Regulation (GDPR). Further requests for custom analysis without access to the raw data (i.e., collaboration) can be made to the corresponding authors per e-mail. The processed single-nucleus sequencing data with associated metadata is available on Zenodo under 10.5281/zenodo.18370645 (https://zenodo.org/records/18370646) (multi-omic dataset) and 10.5281/zenodo.18371192 (https://zenodo.org/records/18371193) (FANS-seq dataset). The publicly available data was accessed from the provided resources in Maynard et al. (49), (50), Pineda et al. (34), Wang et al. (17), Li et al. (56), Limone et al. (62), Liu et al. (10) and Gittings et al. (18). Source data is provided with this study.

Research involving human participants, their data, or biological material

Policy information about studies with human participants or human data. See also policy information about sex, gender (identity/presentation), and sexual orientation and race, ethnicity and racism.

Reporting on sex and gender	Biological sex was listed as reported from the cohort records, validated in silico from the sequencing data and referred to as 'sex'. No data was available on gender from the cohort records. Individual-level sex is provided with the data for each sample.
Reporting on race, ethnicity, or other socially relevant groupings	No race, ethnicity of other socially relevant data was collected relevant to this study.
Population characteristics	The study includes 82 samples in two datasets: the multi-omic dataset and the FANS-Seq dataset. 72 samples are unique for the multi-omic dataset, 7 are found in both datasets, and 3 are unique to the FANS-Seq dataset. Samples were collected at 6 different centers: Boston (6 Samples), Edinburgh (22 Samples), Hannover (11 Samples), NBB (33 Samples), Ulm (8 samples) and Ulm-2 (2 Samples). The cohort/center information for the samples in the FANS-Seq dataset is not specified to preserve privacy (privacy concerns due to small sample size).<br><br>The multi-omic dataset is comprised of 79 samples in the age range 33-96 years (mean: 66.53, median: 65.5, IQR: 56.25-77.75). One sample had missing age information. Age distribution was significantly different between ALS and Control groups and not significant between ALS-FTD and Control groups and between ALS and ALS-FTD groups (Wilcoxon rank sum test with alpha=0.05). Male/female distribution in the whole cohort was 61%/39% and not significantly enriched in any case group (Fisher's Exact Test p-val > 0.05).<br><br>The FANS-Seq dataset is comprised of 10 samples. After sorting and sequencing, TDP-43-High nuclei were available from 10 of these samples and TDP-43 Low nuclei from 8 of these samples. The age range was 60-75 years (mean: 67.38, median: 66.50, IQR: 64.0-70.75). Age distribution was not significantly different between the TDP-43-High and TDP-43-Low groups (Wilcoxon rank-sum test with alpha = 0.05; groups ages are not identical because from two sample donors only TDP-43-High nuclei were successfully sorted and sequenced). Male/female distribution in the whole cohort was 70%/30% and not significantly enriched in one of the TDP-43 High/Low groups (70%/30% & 62.5%/37.5%, Fisher's Exact Test with alpha 0.05).<br><br>Due to the heterogeneity of data collected at the different centers comprising the study cohort, more comprehensive population data could not be collected to be used for covariate modeling. Therefore, covariates were extensively modeled with surrogate variable analysis as indicated in the manuscript.<br><br>Genetic background relevant to the diagnosis was tested for mutations in a panel of 43 ALS-related genes as described in the methods section.
Recruitment	Participants were recruited through the respective post-mortem tissue collection programs at each center. At each center, donation was voluntary and altruistic and participants did not receive financial compensation for their participation.
Ethics oversight	The research approach has been approved by the Ethics Committee of the University of Ulm 119 (Nr. 135/20-FSt/TR).<br>All material has been collected from donors for or from whom a written informed consent for a brain autopsy and the use of the material and clinical information for research purposes had been obtained.

Note that full information on the approval of the study protocol must also be provided in the manuscript.

Field-specific reporting

Please select the one below that is the best fit for your research. If you are not sure, read the appropriate sections before making your selection.

☒ Life sciences ☐ Behavioural & social sciences ☐ Ecological, evolutionary & environmental sciences

For a reference copy of the document with all sections, see nature.com/documents/nr-reporting-summary-flat.pdf

Life sciences study design

All studies must disclose on these points even when the disclosure is negative.

Sample size	Sample sizes were estimated from previous studies and recommendations for genomics studies. No statistical method was used to predetermine sample size.
Data exclusions	No samples were excluded. Vascular and immune cells were excluded due to low numbers that prevent proper analysis.
Replication	Multiple samples from different centers were included in the study cohort. Raw data, processed data and data analysis code are provided with this study to increase reproducibility.
Randomization	Samples were allocated to the case groups based on diagnosis.
Blinding	Blinding was not possible during sample collection, as specific, matched samples had to be selected and pooled together. The investigators were blinded to group allocation during sample preparation, sequencing, and pre-processing of the data during data analysis. Blinding of the

data analysts was not relevant for the downstream data analysis, as it is performed computationally, based on analysis statistics, and not by subjective judgement of the investigators.

Reporting for specific materials, systems and methods

We require information from authors about some types of materials, experimental systems and methods used in many studies. Here, indicate whether each material, system or method listed is relevant to your study. If you are not sure if a list item applies to your research, read the appropriate section before selecting a response.

Materials & experimental systems

n/a	Involved in the study
☐	☒ Antibodies
☒	☐ Eukaryotic cell lines
☒	☐ Palaeontology and archaeology
☒	☐ Animals and other organisms
☒	☐ Clinical data
☒	☐ Dual use research of concern
☒	☐ Plants

Methods

n/a	Involved in the study
☒	☐ ChIP-seq
☐	☒ Flow cytometry
☒	☐ MRI-based neuroimaging

Antibodies

Antibodies used

Anti-TDP-43 (1:500), CoraLite488-conjugated TDP-43, #CL488-10782, Proteintech
Anti-NeuN (1:500), Milli-Mark™ Anti-NeuN-PE antibody, #FCMAB317PE, Sigma-Aldrich

Validation

Anti-TDP-43 (1:500), CoraLite488-conjugated: Antibody validated by the manufacturer: "Ko/Kd Validated", Antigen affinity purification. https://www.ptglab.com/products/TDP-43-Antibody-CL488-10782.htm?srsltid=AfmBOopcSZdTXiWQn52x-KxdDVAb5H7aSQXSbEgk8HBSmBIJR9jzKTyt

Anti-NeuN (1:500), Milli-Mark™ Anti-NeuN-PE: Antibody validated by the manufacturer: "This Milli-Mark Anti-NeuN-PE Antibody, clone A60 is validated for use in FC for the detection of NeuN.". Clone A60.
https://www.sigmaaldrich.com/DE/en/product/mm/fcmab317pe?srsltid=AfmBOorcPvykuqXy4-YnkmrSPzKFWfasWlooa2hoCKWQcizfojSx1Of_

Plants

Seed stocks

N/A

Novel plant genotypes

N/A

Authentication

N/A

Flow Cytometry

Plots

Confirm that:

☒ The axis labels state the marker and fluorochrome used (e.g. CD4-FITC).

☒ The axis scales are clearly visible. Include numbers along axes only for bottom left plot of group (a 'group' is an analysis of identical markers).

☐ All plots are contour plots with outliers or pseudocolor plots.

☒ A numerical value for number of cells or percentage (with statistics) is provided.

Methodology

Sample preparation

The complete sample preparation is described in the 'Methods' section under 'Fluorescence-activated nuclei sorting (FANS)". In short, ~300 mg of frozen primary human brain motor cortex tissue was homogenized, centrifuged on an OptiPrep gradient

and washed. Nuclei were then incubated in 1 ml staining buffer for 15 min on ice and stained in staining buffer for 90 min at 4 °C on a rotating wheel. Nuclei were then centifuged and re-suspended in 500 µL staining buffer for the flow-cytometry nuclear sorting.

Instrument	FACS Aria II SORP (BD) & FACS Aria III (BD).
Software	FACSDiva Version 9.0.1
Cell population abundance	Both collected fractions constituted ~20-25% of all events together. The NeuN+, TDP-43-High fraction constituted >90% of the final collected events.
Gating strategy	The gating strategy is exemplified in Suppl. Fig. 20. In the first gate (P1), nuclei were selected against debris based on SSC-A & FSC-A. In the second gate (P2), singlets were selected from in the FSC-A/FSC-H plot. In the third gate (P3), singlets were selected a second time from the SSC-A/SSC-H plot. DAPI-positive nuclei were then selected in the 4th gate (DAPI+) based on the DAPI signal (>10^4 & < 10^5). The sorting gates were selected from the DAPI+ gate based on the NeuN-PE-A/TDP-43-Coral_Lite_B530_A plot such that both fractions are selected with > 3.10^2 NeuN-PE-A signal.

☒ Tick this box to confirm that a figure exemplifying the gating strategy is provided in the Supplementary Information.
